# Microeconomic adaptation to severe climate disturbances on Australian coral reefs

**DOI:** 10.1007/s13280-022-01798-w

**Published:** 2022-11-02

**Authors:** Henry A. Bartelet, Michele L. Barnes, Graeme S. Cumming

**Affiliations:** ARC Centre of Excellence for Coral Reef Studies, James Cook University, Townsville, 4811 Australia

**Keywords:** Adaptive responses, Climate change, Coral bleaching, Great Barrier Reef, Reef tourism, Social-ecological resilience

## Abstract

Coral reefs are increasingly affected by climate-induced disturbances that are magnified by increasing ocean temperatures. Loss of coral reefs strongly affects people whose livelihoods and wellbeing depend on the ecosystem services reefs provide. Yet the effects of coral loss and the capacity of people and businesses to adapt to it are poorly understood, particularly in the private sector. To address this gap, we surveyed about half (57 of 109) of Australian reef tourism operators to understand how they were affected by and responded to severe impacts from bleaching and cyclones. Reef restoration and spatial diversification were the primary responses to severe bleaching impacts, while for cyclone-impacts coping measures and product diversification were more important. Restoration responses were strongly linked to the severity of impacts. Our findings provide empirical support for the importance of response diversity, spatial heterogeneity, and learning for social-ecological resilience.

**Supplementary Information:**

The online version contains supplementary material available at 10.1007/s13280-022-01798-w.

## Introduction

Coral reefs are one of the first, and probably most iconic, victims of climate change. The Intergovernmental Panel on Climate Change (IPCC) is highly confident that almost all tropical coral reefs will suffer significant losses even if global warming is limited to 1.5 °C (Pörtner et al. 2022). Given that the world will most likely exhaust the 1.5 °C carbon budget before the year 2030 (DNV 2021), it is highly probable that extractive and service industries that depend on healthy coral reefs will be severely affected over the coming decades.

Coral reefs have already come under severe threat from elevated water temperatures and changes in disturbance regimes (Hughes et al. 2018; Goreau and Hayes 2021). For example, the Great Barrier Reef (GBR) in Australia has been affected by mass coral bleaching events in 1998, 2002, 2016, 2017, 2020, and 2022, and has suffered substantial impacts from 10 category-three or higher cyclones between 2004 and 2018. Both the frequency and severity of coral bleaching (Hoegh-Guldberg 1999; Lough et al. 2018) and tropical cyclones (Kossin et al. 2020) are driven by increasing sea temperatures and can lead to significant loss of coral reefs. Rapid degradation of coral reefs has implications for local resource users (Ostrom 2009; Cinner et al. 2013) and consequently has wider socio-economic ramifications. To understand these, better theoretical frameworks and more information are needed about how resource users are impacted by, and are adapting to, the loss of coral reefs (Pendleton et al. 2016; Comte and Pendleton 2018; Hoegh-Guldberg et al. 2019; Stoeckl et al. 2021).

Scholarly research on human adaptation to climate change has been steadily increasing, although most studies remain focused on intended adaptation to future climate change rather than actual adaptation to experienced climate impacts (Berrang-Ford et al. 2021; Bartelet et al. 2022a). Research on adaptation to actual climate impacts by microeconomic actors, specifically in the private sector, also remains limited (Linnenluecke et al. 2013; Fankhauser 2017; Berrang-Ford et al. 2021). A recent framework was developed, based on a review of empirical evidence, stating the hypothesized primary responses microeconomic actors (i.e. households and firms) might take in response to impacts from climate change (Bartelet et al. 2022a). Within the microeconomic adaptation literature, empirical research on adaptation to experienced climate effects remains skewed towards farming (Fankhauser 2017; Bartelet et al. 2022a). Coral reef social-ecological systems provide an excellent case study in which to address two specific research gaps: (1) a lack of information outside agriculture on adaptation to experienced climate change; (2) the responses to climate change by actors in the private sector.

The degree of adaptation evidenced by resource users in response to coral degradation and loss will depend partially on the range and diversity of adaptive responses that are available to them (Norberg and Cumming 2008; Schindler et al. 2015; Grêt-Regamey et al. 2019). Understanding which adaptive responses are available to resource users is the first step in understanding how users may respond, and how these responses may impact the broader response of the social-ecological system as a whole. While a scenario in which resource users do not adapt at all to a changed environment (Easterling et al. 1992; Hoegh-Guldberg et al. 2019) is unlikely, adaptation to the coral reef crisis requires local-scale adaptation to a global driver, making local mitigation of impacts difficult (Cumming et al. 2006). Indeed, the degree to which viable adaptation in this context is possible remains to a large extent unknown.

The degradation of coral reefs will affect the tourism industry in a direct and immediate way. For example, the increasing trend in visitor numbers to the GBR in Australia levelled off after the severe bleaching event in 2016 and visitor numbers started a slow decline thereafter (Bartelet et al. 2022b). Prior studies on adaptation to climate impacts on coral reefs by resource users in the tourism industry have mainly been scenario-based rather than empirical (Biggs 2011; Biggs et al. 2012b; Evans et al. 2016). Business planning, diversification, and stewardship measures were identified as potential adaptation options (Evans et al. 2016), while some tourism operators indicated that they would consider exiting the reef tourism industry under scenarios of reductions in visitor numbers ranging from 10 to 50% (Biggs 2011; Biggs et al. 2012b).

To address the gap in existing knowledge about adaptation strategies in coral reef social-ecological systems, we undertook an exploratory study to empirically assess adaptation to severe climate disturbances on Australian coral reefs by tourism operators. We focused on four primary research questions: (1) how did tourism operators in Australia respond to severe climate-related disturbances, specifically the coral bleaching events in 2016 and 2017 and severe cyclones in 2011 and 2017? (2) How applicable is the microeconomic adaptation framework developed by Bartelet et al. (2022a) towards adaptation to climate change by coral reef tourism operators? (3) Did increasingly severe impacts reduce the adaptation alternatives that were available (Hoegh-Guldberg et al. 2019)? And (4) how did the contextual characteristics of the business affect the adaptation process?

### Background and study sites

We focus our inquiry on coral reef tourism sites in Australia. Our most notable sites are located in the Great Barrier Reef (GBR), the world’s largest coral reef ecosystem covering 344,400 km^2^ along the east coast of Queensland in Australia (GBRMPA 2012). The GBR directly contributed an estimated $6.4 billion in economic value and 64,000 jobs to the Australian economy in the year 2016, of which $5.7 billion (90%) was provided either directly or indirectly by tourism (Deloitte Access Economics 2017). Bleaching events in 2016 and 2017 were followed by coral mortality and significant losses in coral cover along the Central and Northern two-thirds of the Great Barrier Reef, also affecting some of the primary reef tourism locations (GBRMPA 2017; AIMS 2018), although there have been indications of reef recovery in recent years (AIMS 2022). More localized reef areas have also been severely affected by severe tropical cyclones, most notably Cyclone Yasi in 2011 (affecting the area around Mission Beach) and Cyclone Debbie in 2017 (affecting the Whitsunday Islands). We complemented our GBR sites with data from reef tourism operators from other smaller coral reef ecosystems in Australia, specifically the Moreton Bay Marine Park (southern Queensland), the Lord Howe Island Marine Park (New South Wales), Ningaloo Marine Park (Western Australia), and the Cocos Islands Marine Park (Western Australia). Our study thus included reef tourism operators from all around Australia.

## Materials and Methods

### Sampling

We sought to represent the full population of in-water reef tourism operators in Australia that offered recreation-based activities like diving and snorkeling that are directly linked to coral reefs. These operators were identified through an online search (i.e. Google search engine, Google Maps and TripAdvisor) with the search terms “coral tours”, “coral reef tours”, “reef diving”, “reef snorkeling”, and a term for the location (we used both the name of the marine park and the name of the main reef tourism locations, e.g. Cairns). Scenic flight operators and fishing charters were excluded because their visitors do not interact as closely with coral reefs during their tours compared to visitors undertaking in-water activities. We included dive resorts and private charter boats as their visitors often directly interact with coral reefs underwater. For our main sample locations in the GBR Region, we cross-verified our list with reef tourism operators through in-person visitations of the reef tourism areas. We identified a total of 109 reef tourism companies in Australia that were in operation during the specific climate disturbances we studied, e.g. we did not include operators that started their business post-2016 for our bleaching samples. Online Resource 1 provides an overview of the reef tourism operators that were identified through this process. 

Our analytical design included *a priori* treatment and control groups of tourism operators, based on whether their reef sites had been directly affected by a specific climate disturbance (Table 1). Our main focus was on reef operators affected by coral bleaching (treatment), while we included operators that were not affected by bleaching as a control group. We added a second treatment sample focused on operators affected by cyclone impacts to test whether these responses differed from bleaching. We included a question about disturbance severity in our surveys to check whether the treatment/control divide was consistent with our operators’ own experiences. The *posterior* treatment/control divide differed slightly from our *a priori* assumption. One of the 18 reef tourism operators, which was located in the Ningaloo Marine Park MA, that we included in our *a priori* control sample had to be included in the *posterior* treatment group because their reef sites had been directly affected by severe bleaching in 2017. Five of the 39 reef tourism operators in our *a priori* treatment group, four of which located in the Cairns/Cooktown MA and one in the Townsville / Whitsunday MA, had to be included in the *posterior* control group because none of their reef sites had been severely affected by bleaching in either 2016 or 2017. Operators that did not experience direct ecological impacts might still have been affected by reputational effects, or have undertaken other kinds of potentially adaptive responses, and thus our control group surveys provided insights into responses to the indirect or non-ecological impacts of climate-related disturbances.

**Table 1:** Overview of study locations and participation fractions

Marine Park Management Area (MPMA)	State	Reef tourism locations	Sample size (fraction of companies in MPMA)
Cairns/Cooktown Management Area (*a priori* treatment sample)	Queensland (Great Barrier Reef)	Cape Tribulation; Port Douglas; Cairns; Mission Beach	22 out of 39 (56%)
Townsville/Whitsunday Management Area (*a priori* treatment sample)	Queensland (Great Barrier Reef)	Orpheus Island; Townsville; Magnetic Island; Alva; Airlie Beach; Hamilton Island; Daydream Island	17 out of 29 (59%)
Mackay/Capricorn Management Area (*a priori* control sample)	Queensland (Great Barrier Reef)	Yeppoon; Great Keppel Island; Pumpkin Island; Lady Elliot Island; Bundaberg	4 out of 11 (36%)
Moreton Bay Marine Park (*a priori* control sample)	Queensland	Sunshine Coast; Moreton Island; North Stradbroke Island; Brisbane; Gold Coast	5 out of 9 (56%)
Lord Howe Island Marine Park (*a priori* control sample)	New South Wales	Lord Howe Island	2 out of 5 (40%)
Ningaloo Marine Park & Cocos (Keeling) Islands Marine Park (*a priori* control sample)	Western Australia	Coral Bay; Exmouth; West Island (Cocos)	7 out of 16 (44%)

For our bleaching treatment group, we used data from tourism operators in the ‘Cairns / Cooktown’ and the ‘Townsville/Whitsunday’ Management Areas (Table 1) because these areas were most severely affected by the coral bleaching events in 2016 and 2017 (GBRMPA 2017; Hughes et al. 2017; AIMS 2018). We did not include reef tourism operators in the Whitsundays region in the bleaching sample, as they were affected by another severe climatic disturbance (Cyclone Debbie) in 2017, the same period when the bleaching events occurred. For our cyclone treatment group, we focused on tourism operators in the Whitsundays for Cyclone Debbie (2017) and in Mission Beach for Cyclone Yasi (2011).

For our bleaching control group, we focused on tourism operators in the southern sections of the GBR (‘Mackay/Capricorn Management Area’) because these areas were least severely affected by the coral bleaching events in 2016 and 2017. We also approached reef tourism operators from all other coral reef ecosystems in Australia (Table 1) as part of our control group.

All operators were initially invited through e-mail and were later followed up on through either in-person visits or phone calls. About half (57 out of 109) participated in our survey (Table 1) and one operator participated in both the bleaching and cyclone (2011) survey giving us a total sample size of 58. In our treatment samples we reached participation rates nearby 60%. Frequent reasons for not-participating in our survey were (1) no staff around from that time; (2) changed ownership; (3) no time available; and (4) some companies were (temporarily) out of operation due to the COVID-19 pandemic. We used online surveys with company representatives, undertaken with Kobotoolbox survey software, to collect data on adaptive responses and contextual factors. Because we were interested in adaptive response to climate disturbances that occurred before our study, the data we collected were based on recall. Data were collected between October 2020 and July 2022.

### Disturbance and company characteristics

We examined contextual information on the disturbance, the reef tourism business, and the business representative that we hypothesized could be related to how reef tourism operators responded to climate disturbances (Table 2).

**Table 2:** Explanatory variables for adaptive response models to climate disturbances. Categorization of business size was done based on three clusters that were identified in collected data

Variable	Description	Data type	Unit of measurement
Disturbance type	Type of climate disturbance to which the reef tourism operators had to adapt	Binary	(0) Bleaching (1) Cyclone
Disturbance severity	Fraction of reef sites used on tours before disturbance that had more than a third of their area affected by climatic impact (either bleached or damaged by cyclone)	Continuous	(0) None of reef sites (1) 25% of reef sites (2) 50% of reef sites (3) 75% of reef sites (4) All of reef sites
Business type	Fraction of customers that engaged in scuba diving versus snorkelling activities	Binary	(0) Mostly snorkelling (1) Mostly scuba
Business size	Total number of passenger seats on company’s boats as proxy for business size	Categorical	(1) Small (0–20 seats) (2) Medium (20–200 seats) (3) Large (> 200 seats)
Age	Age group of the company representative (respondent in our survey)	Binary	(0) Above 45 years (1) Below 45 years
Gender	Gender of the company representative (respondent in our survey)	Binary	(0) Male (1) Female

We included the disturbance type and severity as the main distinguishing characteristics of the disturbance. We distinguished between bleaching and cyclones because we expected a different qualitative and quantitative nature of these impacts. Bleaching can destroy reefs, but there is a time lag of years between when a reef is bleached and when its fish biomass declines (if the reef does not recover), whereas cyclones can turn reefs to rubble in a few hours, although the effects are more patchy (Cheal et al. 2017; Dietzel et al. 2021). Disturbance severity measures the spatial severity of the climate disturbance for a particular operator in terms of what fraction of the reef sites they were using were severely affected. Prior studies have argued that the severity of impacts on coral reefs might affect the availability of adaptation alternatives for tourism operators, for example their ability to relocate to healthy reef areas (Hoegh-Guldberg et al. 2019; Stoeckl et al. 2021). We followed previous research that identified severe bleaching as more than a third of a reef being affected (Hughes et al. 2018). For locations where we studied adaptive responses to two consecutive bleaching events, e.g. GBR 2016 and 2017, we asked for disturbance severity for each year separately and used the highest severity value as a predictor in our models.

We accounted for the business and business representative characteristics (Running et al. 2019) by including the business type (scuba diving versus snorkelling) and size as well as the age and gender of the company representative. We used the business type variable as a proxy for the company’s customers’ sensitivity to coral conditions. Here we hypothesized that reef tourism operators catering more towards snorkelling than diving would have visitors that are, on average, less knowledgeable about reef conditions and thus less sensitive to impacts from climate disturbances (Leujak and Ormond 2007; Uyarra et al. 2009). On the other hand, snorkelling sites are usually shallower and these sites might have been more severely affected by the climate impacts, as measured through our control factor of disturbance severity. The business size was found to be an important determinant of adaptation in farming settings (Bartelet et al. 2022a). For example, households with larger farms were more likely to diversify within their livelihood, to manage natural resources, and to change their mode of operating. They were less likely to diversify between livelihoods. We measured the number of passenger seats using nine multiple-choice options that ranged from ‘0–10 seats’ to ‘ > 500 seats’. Through visual inspection of the data, we identified three clusters that we consequently categorized as small, medium, and large. We included company size as a categorical rather than an ordinal predictor because the effects were not ordered linearly for all response models.

Inclusion of the age group of the company representative was based on prior findings in a farming setting,where the age of the head of the household was a significant predictor for several adaptive responses (Bartelet et al. 2022a). Specifically, younger age increased the likelihood of diversification between livelihoods, changes in the mode of operating and the management of natural resources, while reducing the likelihood of diversification within livelihood and protective measures. The effect of gender on adaptation has been acknowledged as a research gap, although so far there have been few specific hypotheses regarding its linkage to particular adaptation behaviors (Bunce and Ford 2015; Mortreux and Barnett 2017). One study found that within rural households in Australia, women are less likely to be involved in adaptation to wildfire due to low empowerment (Eriksen et al. 2010). Given that our dataset included formal tourism businesses, we hypothesized that the gender of the representative might be less of a barrier as compared to rural households, but acknowledge that any existing power differentials along the lines of gender could potentially have affected adaptive responses.

### Adaptive responses to climate disturbances (*a priori* classification)

Because of the lack of empirical knowledge on adaptation to climate change by coral reef tourism operators, we used an exploratory approach to identify the generic response types that were adopted. Our classification was based on six generic types of adaptation identified in the framework by Bartelet et al. (2022a) which were found to be commonly implemented in response to actual climate change impacts by other microeconomic resource users, most notably farmers (Table 3). Within these six types of adaptation, we defined a number of more context-specific adaptive responses based on the tracking of adaptation measures as described in the written media, through expert consultation, and through pilot interviews. Through this process we also added a novel adaptive response linked to ‘climate action’, i.e. reef tourism operators becoming involved in measures to reduce carbon emissions.

**Table 3:** Microeconomic adaptive responses to climate disturbances on coral reefs, based on the framework by Bartelet et al. (2022a) which identified the most common responses by other microeconomic actors in response to climate change. Adaptive responses sorted by the frequency of observance in other microeconomic settings. We added ‘climate action’ as an additional response specifically for the (reef) tourism sector

Type of adaptation	Adaptive responses	Description
Diversification within livelihoods	(1) Spatial diversification (2) Product diversification	Changing reef sites company was visiting on tours Changing the type of tours or activities company was offering to tourists
Operational change	(3) Making changes to the way the company is running its day-to-day operations	Changing logistics (e.g. tour season), personnel, sales (e.g. price change), and/or marketing
Natural resource management	(4) Reef restoration	Enacting or participating in measures to improve the health of the coral reef
Diversification between livelihoods	(5) Switching livelihood activities entirely or partly	Diversifying to products/services outside of tourism
Reduction of immediate impact (relief)	(6) Relief measures (7) Support-seeking	Selling of property (e.g. boats, equipment and/or office space), reduction of workforce, and/or relying on savings Seeking support from government, local community, and/or relatives
Protection of livelihood	(8) Risk protection (9) Monitoring	Seeking or purchasing protection from risks (e.g. insurance) Beginning monitoring climate and/or reef conditions
Climate action	(10) Carbon dioxide (CO_2_) reductions	Enacting or participating in measures to reduce CO_2_ emissions of company, customers, and/or community

Respondents were asked: (1) whether they had used each of the ten particular adaptive responses; (2) whether they had implemented any response that was not included in our list; and (3) to select their most important (primary) response to the climate disturbance out of all responses taken. For GBR operators that were affected by two consecutive bleaching events, we asked respondents for responses that were implemented over the period March 2016 to March 2018. For GBR operators that were affected by Cyclone Debbie in 2017, we asked for responses over the period March 2017 to March 2018. We decided to use an adaptation period of one year after a disturbance because using a longer time period would make it harder to attribute responses to specific climate events rather than other causes.

After collecting data on which of the ten adaptive responses were used by each operator, we used partial correlation analysis to identify which responses most often clustered together and tested for Spearman’s Rank correlation using the ‘ppcor’ package (Kim 2015) in R software (R Core Team 2013). Based on the data, the categorization of six adaptation types found from other microeconomic settings (Bartelet et al. 2022a), and based on our contextual understanding of the reef tourism system we merged some of the responses into combined response categories. The response clustering as presented in Table 3 should therefore be considered as an *a priori* classification that will be updated based on the empirical results from our case study with Australian reef tourism operators.

### Analysis

Our response variables were classified as binary (i.e. used or not used). We therefore used logistic regression models to analyse the effect of the predictors on the likelihood of implementing a particular adaptive response. Modelling was done in R software using the generalized linear models (*glm*) function. Code is available in R Markdown (Online Resource 2) and we provided the input data in Online Resource 3. We standardized our non-binary predictor (disturbance severity) using z-scores, by subtracting the mean and dividing by twice the standard deviation (Gelman 2008). Dividing by twice the standard deviation standardizes a variable to have a mean of ‘0’ and a standard deviation of ‘0.5’; this technically standardizes the variable on a binary scale. The coefficient for our disturbance severity predictor is now directly comparable and should be interpreted as the effect of a one-standard deviation change in the predictor variable on the response variable. All predictors had a variance inflation factor (VIF) below 4, indicating low collinearity in our models. The models were validated via DHARMa residuals (Hartig 2018). Inferences were based on a 95% significance level. 

## Results

### Adaptive responses to climate disturbances (*posterior* classification)

**Fig. 1:**
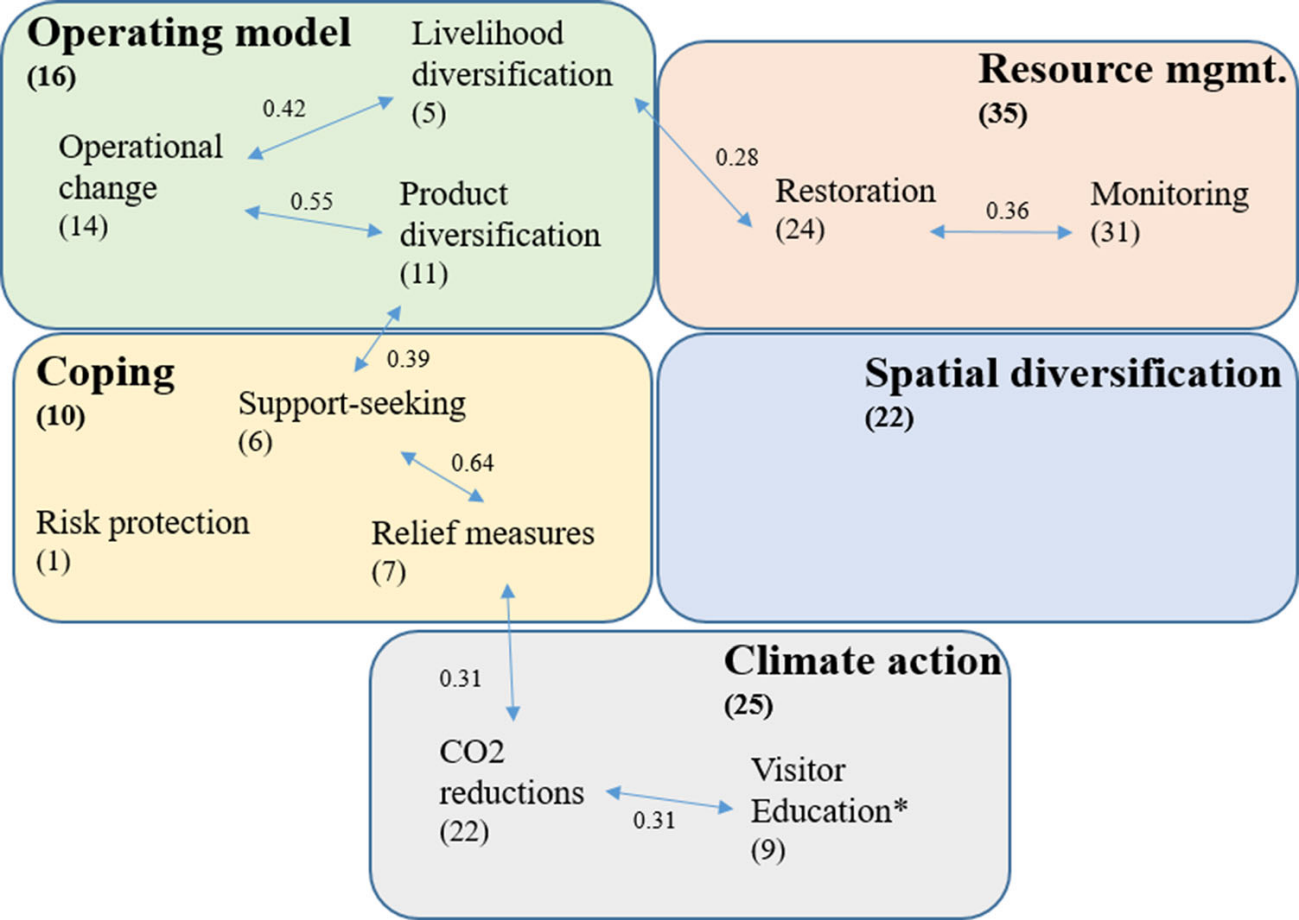
Clustering of adaptive responses to climate disturbances by Australian reef tourism operators (*n* = 58). Graph includes only significant partial correlation effects (Spearman’s rank correlation coefficient) between responses that are significant at a *p*-level of 5%. Numbers in brackets indicates prevalence of adaptive response (i.e., how many operators adopted a particular response). We decided to include risk protection within the ‘Coping’ cluster because it was only used by one operator in the sample and we conceptually judged it to be most applicable to this cluster. Visitor education was mentioned as ‘other’ response by nine operators in our sample (16%) and we merged this response within the climate action cluster because it was significantly correlated with actions to reduce carbon dioxide (CO_2_) emissions

We found eight positive partial correlations between our individual adaptive responses that were significant at a p-level of 5% (Fig. 1). Based on these significant associations, we decided to make some changes to the *a priori* classification of adaptive response as proposed in Table 3. Most notably we decided to merge the adaptive responses of operational change, product diversification, and livelihood diversification into a combined adaptive response cluster linked to changes in ‘operating model’ because they were all linked to responses on the business and operational side. Compared to our *a priori* categorization, we classified ‘spatial diversification’ as a separate adaptation cluster because it was frequently implemented and not significantly associated with any of the other adaptive responses.

We found that the adaptive responses of ‘monitoring (reefs and/or climate)’ and ‘restoration’ were significantly correlated, although our *a priori* classification had defined monitoring as a protective measure. We used the monitoring and restoration responses as separate responses in our consequent analysis because these were each implemented by a relatively large fraction of operators. In accordance with our *a priori* classification, the adaptive responses of ‘relief measures’ and ‘support-seeking’ were significantly correlated.

Finally, one of the adaptive responses that was mentioned as other response by 16% of the participants was ‘visitor education’, i.e. informing and educating visitors about the causes and consequences of the climate disturbances. We merged the visitor education response with ‘climate action’ because they were significantly associated and because visitor education could potentially have an effect on future carbon emissions similar to a company taking climate action itself.

### Adaptive responses to coral bleaching

**Fig. 2:**
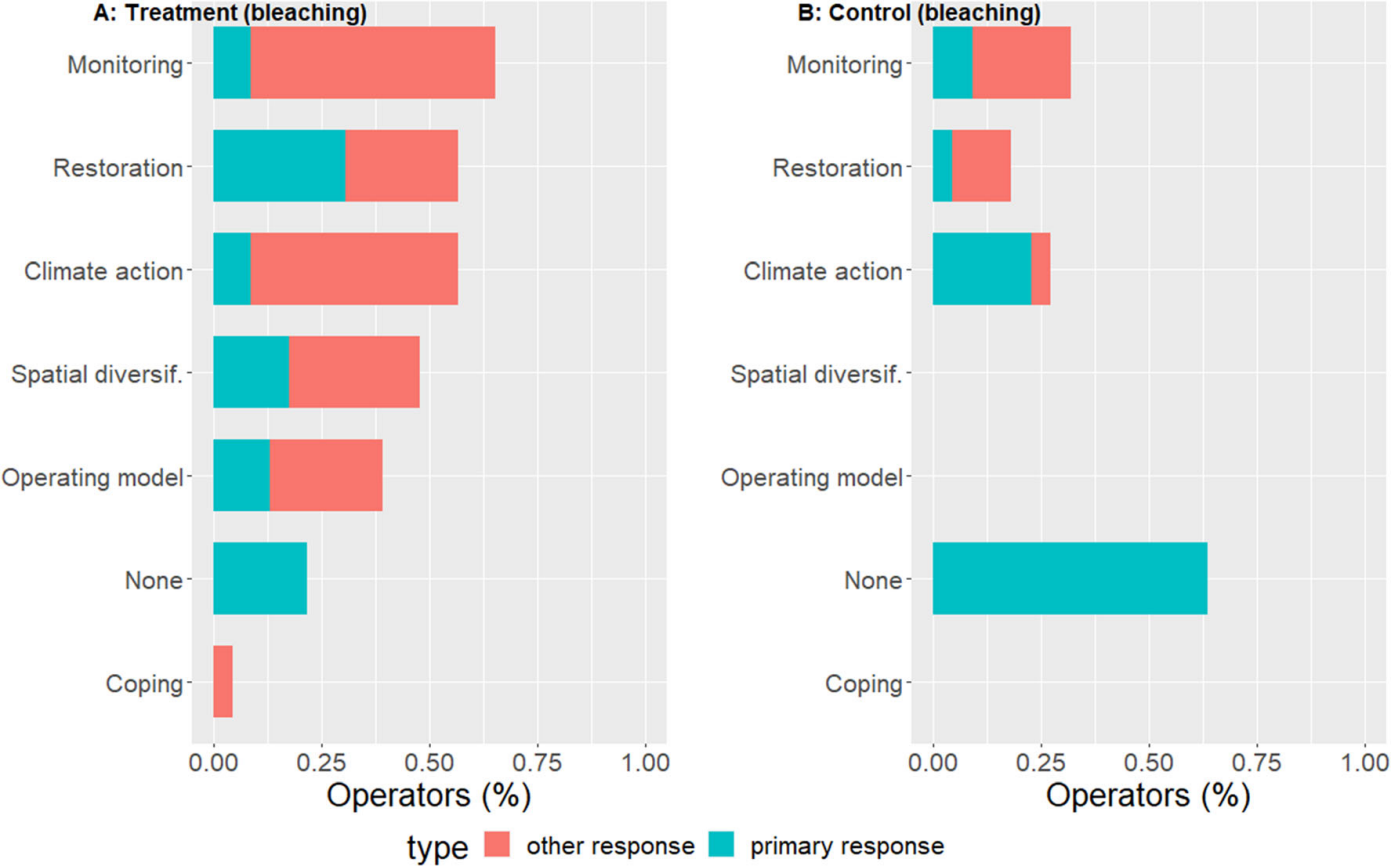
Adaptive responses to coral bleaching impacts in Australia. Graph (**A**) shows responses by reef tourism operators that had at least 25% of their reef sites severely affected by the climatic disturbance (*n* = 23). Graph (**B**) shows the proportion of reef operators who took similar action in response to bleaching but were not directly affected by it (i.e., the control group, where none of their reefs were severely affected) (*n* = 22). Climate action includes visitor education; operating model includes changes in the mode of operating, product diversification, and livelihood diversification. Coping measures includes relief measures, support-seeking, and seeking protection from risks

GBR tourism operators in our treatment sample implemented a wide variety of adaptive responses to impacts from coral bleaching (Fig. 2A), while responses by operators in our control group were less diverse and common (Fig. 2B).

In our treatment sample, 22% of the reef tourism operators did not implement any adaptive response to the impacts from coral bleaching. The most common response to bleaching impacts was to begin with monitoring climate and/or reef conditions, while enacting or participating in measures intended to improve the health of the coral reef (i.e., restoration measures) was most often mentioned as the primary response. Climate action and spatial diversification were implemented in response to bleaching impacts by about half of the sampled operators in the treatment sample. The majority (64%) of operators in our control group did nothing as primary response to the disturbance that did not directly affect their reef sites, while 23% took climate action as primary response. None of the operators in the control sample spatially diversified their reef sites and/or changed their operating model in response to bleaching. Operators in our treatment group were twice as likely to take climate action in response to coral bleaching as compared to operators in the control group (57% versus 28%).

### Adaptive responses to tropical cyclones

**Fig. 3:**
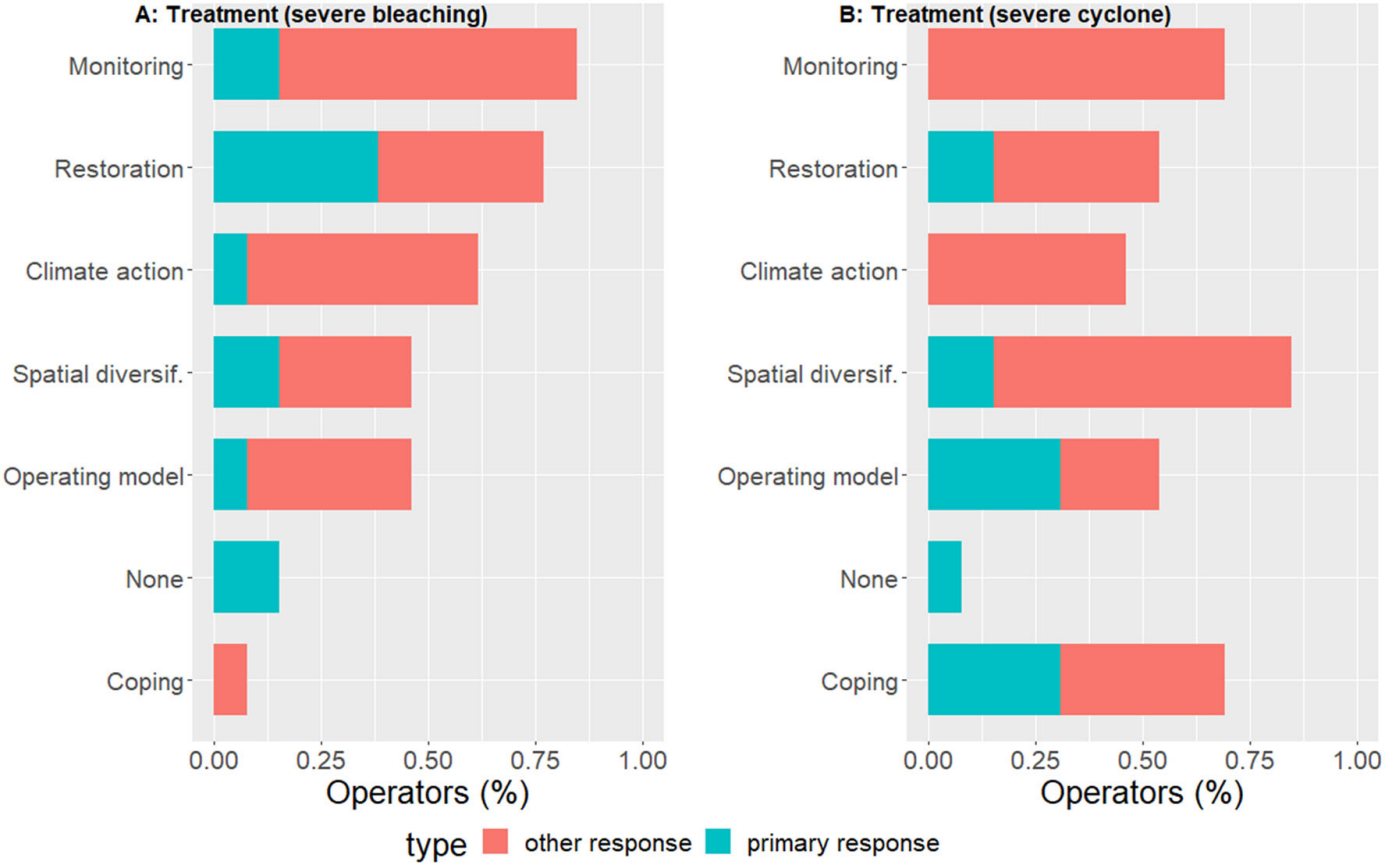
Adaptive responses to severe coral bleaching and cyclone impacts in Australia. Graph (**A**) shows responses by reef tourism operators that had at least 50% of their reef sites severely affected by coral bleaching (*n* = 13). Graph (**B**) shows responses by reef operators that were affected by cyclone impacts, all of which had at least 75% of their reef sites severely affected (*n* = 13)

Adaptive response to impacts from coral bleaching differed from responses to cyclones (Fig. 3). For cyclone impacts, spatial diversification became the most common adaptive response, while coping measures and changes in the company’s operating model were most often implemented as a primary response. Three out of four of the primary responses within the ‘operating model’ responses for cyclones were linked to product diversification and one to livelihood diversification. Within the coping measures cluster, three out of four of the primary response were linked to relief measures and one to seeking protection from risks.

### Disturbance and company characteristics associated with adaptive responses

About half of the operators in our sample had at least 50% of their reef sites affected by a climate disturbance (Table 4). The majority of respondents were mainly focused on snorkeling activities, had less than 20 passenger seats on their company’s boats, and had a male company representative that was older than 45 years.

**Table 4:** Combined sample description for reef tourism operators affected by bleaching and cyclones

Indicator	Indicator levels	Frequency (sample fraction)
Disturbance type	Bleaching	45 (78%)
	Cyclones	13 (23%)
Disturbance severity (% of reef sites severely affected)	0% of reef sites	22 (38%)
	25% of reef sites	10 (17%)
	50% of reef sites	7 (12%)
	75% of reef sites	8 (14%)
	100% of reef sites	11 (19%)
Business type	Mostly snorkeling	35 (60%)
	Mostly scuba	23 (40%)
Business size (# of passenger seats on company’s boats)	Small (0–20 seats)	28 (48%)
	Medium (20–200 seats)	21 (36%)
	Large (> 200 seats)	9 (16%)
Company representative: Age	Above 45 years	31 (53%)
	Below 45 years	27 (47%)
Company representative: Age	Male	33 (57%)
	Female	25 (43%)

We found six relationships between our predictor variables and the adaptive responses that were significant at a p-level of 5%, four of which were linked to disturbance characteristics and two to the company representative (Table 5). We could not model the response of coping measures as the model did not converge because this response was highly skewed towards the cyclone sample. The models for the adaptive responses of spatial diversification and reef conservation had the highest predictability with respective R-squared values of 0.34 and 0.32.

**Table 5:** Logistic regression statistics for adaptive responses to climate disturbances on Australian coral reefs. Adoption rate reflects fraction of the total sample (*n* = 58) that adopted particular response. R-squared reflects the proportion of the variance in the response variable that could be explained by the predictor variables. Coefficients are on log-odds (logit) scale. Coefficient for disturbance severity is based on z-scored variable to make its effect size comparable to the other binary predictors, and should be interpreted as the effect of a one-standard deviation change in the predictor variable on the response variable. Evidence against the null hypothesis of ‘no effect’ for each predictor is estimated using p-values with a 5% significance level (p-valued provided between brackets)

	Operating model	Spatial diversif.	Monitoring	Restoration	Climate action
Adoption rate	26%	38%	53%	41%	43%
R-squared	0.23	0.34	0.13	0.32	0.16
Disturbance: Cyclone	−0.27 (0.792)	1.11 (0.334)	−0.71 (0.508)	**−2.79** (0.038)*	−1.26 (0.221)
Disturbance severity	**2.82** (0.010)*	1.94 (0.067)	**1.92** (0.044)*	**4.11** (0.003)**	1.26 (0.156)
Business type: Scuba	−0.32 (0.680)	−0.52 (0.499)	0.44 (0.502)	−0.75 (0.336)	0.99 (0.147)
Business size: Medium	−1.25 (0.151)	0.97 (0.226)	0.91 (0.174)	0.62 (0.436)	0.67 (0.340)
Business size: Large	−0.74 (0.484)	−0.03 (0.977)	0.91 (0.317)	1.47 (0.165)	1.06 (0.265)
Representative: Below 45 years	0.17 (0.830)	−0.96 (0.244)	0.06 (0.929)	1.32 (0.141)	**−1.47** (0.036)*
Representative: Female	−0.28 (0.714)	1.02 (0.224)	0.11 (0.864)	**−2.12** (0.013)*	0.08 (0.906)

Bold values are the correlation coefficients that were found to be significant at a p-value of 5%

We found significant evidence against the null hypothesis that the severity of disturbance effects on individual operators would have no effect on the likelihood of three out of five responses being adopted to a climate disturbance: changes in operating model, monitoring, and restoration. Disturbance severity had the strongest effect size on the likelihood of implementing restoration measures. As compared to responses to coral bleaching, operators that were affected by tropical cyclones were more likely to spatially diversify their reef sites and less likely to adopt all other responses. The evidence against the null hypothesis (of no difference between bleaching and cyclone impacts) was significant for restoration measures: this response was significantly less likely for cyclone impacts. We found significant evidence against the null hypothesis that age of the company representative would not affect the likelihood of climate action: Companies with younger representatives were significantly less likely to undertake climate action. Finally, we found evidence against the null hypothesis that the gender of the company representative would not affect the likelihood of restoration responses: Companies with female representatives were significantly less likely to undertake restorative action. 

## Discussion

We explored adaptive responses by reef tourism operators to severe climate disturbances on coral reefs in Australia. We found that climate impacts from coral bleaching and tropical cyclones led to a diverse range of adaptive responses (research question 1). The most common responses included the monitoring of climate and/or reef conditions, reef restoration, spatial diversification, and climate action (Figs. 2 and 3). Overall, a previous classification of adaptive response categories based mostly on farmers affected by climate change (Bartelet et al. 2022a) applied well to adaptation by GBR tourism operators (research question 2). Increasingly severe impacts had an overall positive effect on the diversity of responses that were implemented. However, the impacts from tropical cyclones reduced the likelihood of restoration responses (research question 3). Finally, contextual characteristics of the company representative (age and gender) mediated some of the observed diversity in responses (research question 4).

Our study identified only one common adaptive response that was not included in our survey: the education of visitors about climate impacts. Given that tourism operators are directly interacting with consumers (unlike most farmers), this additional response is likely to be industry-specific. Our results indicate that in the specific case of reef tourism operators the adaptation categories of ‘diversification between livelihoods’, ‘changes in the mode of operating’, and ‘product diversification’ were associated and could be clustered together as one common response focused on making changes to a company’s operating model. While product diversification was conceptually clustered together with ‘spatial diversification’ within the ‘diversification within livelihood’ adaptation category (Table 3), our results (Fig. 1) indicate that spatial diversification might be a qualitatively different adaptation response from other kinds of within-company diversification, and therefore may need to be treated separately. Further empirical research within other settings, such as agriculture, are needed to explore the accurateness of the adaptive response classification used here (Bartelet et al. 2022a). Within an agricultural setting, empirical studies could test whether the changing of crop types and/or varieties is associated with the spatial diversification of farm sites or whether these should be considered as separate types of adaptation.

Reef restoration measures (to improve the health of the coral reef) were most often implemented as the primary, i.e. the most important, response to coral bleaching (this was the primary response for 30% of the treatment sample). This could be interpreted as evidence for resource users seeking to restore service provision as triggered by changes in ecosystems (Chapin et al. 2010, 2022). The likelihood of responding by engaging in reef restoration was strongly affected by disturbance severity; i.e., the effect was larger than that for other adaptive responses. This could indicate that if most touristic reef sites are severely affected by a climate-related disturbance, it may trigger some kind of restoration response (e.g., trying to prevent disturbance to damaged areas and/or facilitating its recovery) by commercial users of the reef. However, the effectiveness of specific restoration responses requires further research as persistent, reoccurring bleaching reduces the reef’s ability to recover because of dead coral skeletons that reduce coral regrowth (Hughes et al. 2019a) and lower levels of stock replenishment (Hughes et al. 2019b). Increased mortality of corals, and the direct destruction of reefs, might explain why reef tourism operators who were affected by cyclone impacts were less likely than operators affected by bleaching to adapt by enacting or participating in measures to improve the health of the coral reef. Cyclone-related damage on coral reefs is likely more severe in the short-term than bleaching-related coral mortality because it often affects not only the coral polyps but also the reef substrate. Tourism operators might consider restoration activities less suitable for impacts from cyclone-damaged reefs. Our findings thus provide some support for the hypothesis that increasingly severe impacts might reduce the adaptation alternatives that are available to resource users (Hoegh-Guldberg et al. 2019). There may also be other mechanisms that contributed to our finding that reef restoration was a less frequent response for operators affected by cyclones. For example, it is highly likely that tourism operators that were affected by cyclone impacts had to deal with additional above-the ground damage (to boats, buildings, and communal tourism infrastructure), which may have provided them with less financial and human resources to participate in measures to improve the health of the coral reef as well.

Spatial diversification was an important adaptive response to climate disturbance on coral reefs as hypothesized by other authors (Hoegh-Guldberg et al. 2019; Stoeckl et al. 2021). About half of the operators relocated to different reef sites on their tours in response to bleaching impacts. This finding could explain why visitor satisfaction on the GBR did not decrease during and after the bleaching events in 2016 and 2017, which was reported in a recent study (Bartelet et al. 2022b). Operators might have temporarily or permanently relocated their tours to other reef sites that were of similar quality to the sites they were using before the disturbance, and thus visitor experiences might have been comparable before and after. Our findings align with other recent empirical studies that identified spatial diversification as key adaptation strategy to environmental change (Pecl et al. 2019; Silas et al. 2020; Gonzalez-Mon et al. 2021; Powell et al. 2022) and as an important response by alpine tourism operators affected by climate change (Hoffmann et al. 2009; Mourey et al. 2020; Welling and Abegg 2021). In our cyclone-impacts sample, about 80% of operators responded by changing their reef sites. This fraction was higher than the 50% of reef tourism operators in our treatment sample that responded spatially in response to bleaching. This difference was mostly explained by impacts from cyclones in our sample being overall more severe than those from coral bleaching. When we accounted for disturbance severity in our models, we did not find cyclone-affected operators to be significantly more likely to spatially diversify their reef sites.

Adaptive responses that were not, or were sparsely used by our bleaching treatment sample were more frequently used by our cyclone-impacts sample. That is, relief measures (e.g. selling assets, reducing staff, etc.), seeking support, and diversification between livelihoods were implemented by respectively 50%, 40%, and 30% of the reef tourism operators in our cyclone treatment sample. Notably, relief measures and the changing of tour activities were most often implemented as the primary response to impacts from cyclones. Thus we found that impacts from cyclones led a significant fraction of resource users to diversify their livelihoods away from their preferred ecosystem (Hoegh-Guldberg et al. 2019; Stoeckl et al. 2021). Our findings complement other empirical research that suggests microeconomic actors are likely to diversify their livelihoods in response to environmental change (Hossain et al. 2018; Barnes et al. 2020). The support-seeking response might be more common for cyclone impacts because of the larger terrestrial impacts, while habituation might also play a role. The Queensland Government (where many of our sites were affected by both bleaching and cyclones) has well-established disaster relief packages for cyclones, but not for bleaching, which could have impacted this result.

Characteristics related to the company representative had a strong effect on the implemented adaptive responses, in particular on reef restoration and climate action. Reef tourism operators that were represented by female respondents were significantly less likely to become involved in reef restoration. Speculatively, this could indicate that companies represented by females might have less confidence or opportunities in restoration-related activities. Further research is required, for example to evaluate whether any gender-related differences exist in perceptions towards restoration and to access to restoration funding and opportunities. Younger company representatives were significantly less likely to take climate action. The lower likelihood of companies represented by younger representatives to take climate action was surprising, as existing research indicates that older people are often more sceptical about climate change (Weber 2016). Speculatively, our findings could indicate a legacy effect (Frumkin et al. 2012) where the companies led by an older generation of leaders want to leave an intact ecosystem for younger generations. Alternatively, younger leaders (and/or companies) might not have the required financial resources to invest in carbon reduction technologies.

More generally, our results provide a clear example of several proposed principles of resilience theory in action (Biggs et al. 2015). Diversity (in the form of spatial heterogeneity in the impacts of disturbance regimes), coupled with the availability of large areas of coral reef, appeared to enhance resilience by allowing operators to choose less-impacted reefs for tourism activities. However it remains unsure whether current adaptive responses enhance longer-term social-ecological resilience. The options for relocating to unaffected sites will become more limited as threats from elevated water temperatures and changes in disturbance regimes will increase. It could thus be argued that current adaptive responses are mainly ‘buying time’ until more robust adaptation and mitigation strategies are being developed and undertaken (Howden et al. 2007; Hallegatte 2009). A substantial number of operators deliberately encouraged learning and participation in reef management, presumably in an effort to enhance reef social-ecological resilience. Whether local restoration efforts will be successful in increasing reef resilience and sustaining the attractiveness of the coral reef ecosystem as a major tourist attraction remains an empirical question for the future. In the case of local reefs that were severely affected by cyclone impacts, our results suggest that reef tourism operators already consider product diversification as a viable adaptation strategy.

The main limitation of our study was the exploratory approach we used to identify the most common and important adaptive responses within a coral-reef tourism setting. While we aimed to identify the most common types of adaptation, further research focusing on studying the most common responses in more detail as well as their social-ecological outcomes would enhance our understanding of adaptation and reef decline. For example, we did not account for the different types of involvement in restoration measures that could range from observation and reporting to active engagement (e.g. in crown-of-thorns starfish (*Acanthaster planci*) control or coral nurseries). Second, our sample might have been biased towards operators that would be more likely to engage in restoration measures as compared to the total population. We found that two common reasons for not participating in our study were that operators had either changed ownership or did not have staff around from the time of the first bleaching event we studied in 2016. Previous research with GBR tourism operators had identified lifestyle values as a key predictor of conservation responses (Biggs et al. 2012a). Companies that have their lifestyles attached to the reef will likely be those that have owners, managers, and/or staff that remain with the companies for longer periods of time. Third, given the relatively small population of reef tourism operators in Australia, we did not have the statistical power to include other relevant company and representative characteristics as predictors in our models, such as: quality of coral reefs used by operator; education and experience level of representative; and membership in environmental society or non-governmental organization.

Our study provides empirical evidence for responses to climate change from actors in the private sector, which was identified as a key research gap in the adaptation literature (Berrang-Ford et al. 2021). Our results indicate that adaptation is widespread within the tourism industry and driven in particular by the experienced severity of effects on individual operators. Adaptation is also commonly implemented in tandem with mitigation measures. Our findings provide insights on the views and actions of tourism operators in response to climate-related disturbances, and thereby help in understanding the role of different actors in curbing and adapting to climate-related threats to coral reefs (Barnes et al. 2022). The importance of restoration and spatial responses has implications for reef-related policy makers, in particular in Australia, because environmental regulations and access permit systems might interfere with these preferred adaptive responses by microeconomic actors. On the other hand, government-led reef monitoring and restoration activities that involve tourism operators might have had a positive effect on the observed frequency of conservation and monitoring responses in our sample. For example, the Great Barrier Reef Marine Park Authority involves reef tourism operators in reef monitoring through the ‘Eye on the Reef’ program and in reef restoration through the ‘Crown-of-Thorns Starfish (COTS) control program’.

Further research could focus on doing comparative research on adaptation by reef operators in other locations (e.g. Caribbean, Coral Triangle, and the Red Sea) and in other industries (e.g. agriculture). Comparative research involving multiple regions with larger underlying populations of tourism operators would enable larger samples to be collected, which would permit testing how different levels of adaptive capacity might influence the adaptation process. Such comparative research could also test whether differences in the likelihood of implementing restoration as an adaptive response are indeed linked to the severity of ecological damage. For example, there might be some level of damage from which restorative adaptation becomes unfeasible, whereby microeconomic actors focus dominantly on spatial adaptation and partial or full livelihood change. Understanding such behavioural thresholds and nonlinear effects in complex systems (Janssen 2002; Sterman 2012), e.g. in coral reef social-ecological systems (Bartelet 2017; Leenhardt et al. 2017), will be increasingly important due to the increasing severity of ecological change that is expected in the coming decades.

## Conclusion

Here we showed that reef tourism operators in Australia are already severely affected by and actively adapting to the impacts from climate change. Prominent responses to climate disturbances such as reef monitoring, restoration, and spatial diversification point towards an intensified relationship between commercial users and the natural resource on which they depend. Australian reef tourism operators are also becoming involved in climate action. For cyclone impacts, as compared to bleaching, product and livelihood diversification become more relevant, and they point towards decoupling from the ecosystem. All adaptive responses became more common as operators were more severely affected, although climate action was already frequently undertaken even by operators that were not directly affected by a particular climate disturbance. The ecological impacts from cyclones that could generally be considered as more severe reduced the likelihood of restoration responses. Our results thus point to potential limitations regarding the ability of microeconomic actors to adapt to more severe impacts on ecosystems. Finally, we found that company representative characteristics mediated some of the observed variety in how different actors adapted to climate disturbances. Our findings provide real-world evidence for how resource users are impacted by, and are adapting to, the loss of coral reefs. Such empirical evidence can contribute to knowledge that can be useful for both on-the-ground actors in the private sector as well as policy makers aiming to design effective policies to facilitate microeconomic adaptation to ecological change. Comparative research within and outside of coral reef ecosystems is needed to facilitate generalization of theories on microeconomic adaptation.

## Supplementary Information

Below is the link to the electronic supplementary material.

Supplementary file1 (xlsx 13.4 kb)

Supplementary file2 (PDF 147 kb)

Supplementary file3 (xlsx 28.0 kb)

## Data Availability

Data and code are provided as Supplementary Information.
