# Supplementary material for: Microeconomic adaptation to severe climate disturbances on Australian coral reefs
Source: Ambio. 2022 Nov 2;52(2):285–99. doi: 10.1007/s13280-022-01798-w (PMC9629752; doi:10.1007/s13280-022-01798-w)
Supplement: Supplementary file 2 — Supplementary file2 (PDF 147 kb) [file 13280_2022_1798_MOESM2_ESM.pdf]

Microeconomic adaptation to severe climate disturbances on Australian coral reefs (Ambio)

Henry Bartelet

2022-10-11

1 Publication details

Journal: Ambio
DOI: 10.1007/s13280-022-01798-w
Authors: Henry A. Bartelet[1], Michele L. Barnes[1], Graeme S. Cumming[1]
Affiliation: [1]ARC Centre of Excellence for Coral Reef Studies, James Cook University, Townsville QLD 4811
Corresponding author: Henry A. Bartelet, henry.bartelet@my.jcu.edu.au

Online Resource 2
R Markdown documentation with data preparation and logistic regression models associated with adaptive responses to climate disturbances on coral reefs by Australian reef tourism operators.
R Markdown can be used in combination with Online Resource 3 (after being converted to a .csv file) to reproduce the findings represented in this study.

2 Preparations

Load the necessary libraries

```
#library(tidyverse) #for data wrangling, includes dplyr and ggplot2
library(tidyverse)    #for data wrangling
library(ggnewscale)   #for plotting
library(performance) #for VIF estimation
library(GGally)    #for plotting graphs
library(ggpubr)    #for combining multiple plots
library(DHARMa)   #for residuals and diagnostics
library(ppcor)      #for estimating partial correlations
```

3 Introduction

We undertook an exploratory study to empirically assess adaptation to severe climate disturbances on Australian coral reefs by tourism operators. We focused on four primary research questions: (1) how did tourism operators in Australia respond to severe climate-related disturbances, specifically the coral bleaching events in 2016 and 2017 and severe cyclones in 2011 and 2017? (2) How applicable is the microeconomic adaptation framework developed by Bartelet et al. (2022a) towards adaptation to climate change by coral reef

tourism operators? (3) Did increasingly severe impacts reduce the adaptation alternatives that were available (Hoegh-Guldberg et al. 2019)? And (4) how did the contextual characteristics of the business affect the adaptation process?

4 Description of method

As described in greater detail in our manuscript, we conducted surveys with representatives of reef tourism companies (operators) in Australia. The surveys requested information on the actions that each operator took in response to a specific climate disturbance and a number of predictors linked to the disturbance, company, and representative characteristics.

5 Read in the data

```
data = read.csv('ESM_2.csv')
glimpse(data)
```

```
## Rows: 58
## Columns: 20
## $ X.             <int> 1, 2, 3, 4, 5, 6, 7, 8, 9, 10, 11, 12, 13, 14, 15, 16, ~
## $ resp_age       <chr> "35 - 44", "35 - 44", "45 - 54", "25 - 34", "55 - 64", ~
## $ resp_gender    <int> 1, 0, 0, 1, 0, 0, 1, 0, 0, 1, 0, 0, 0, 0, 0, 1, 0, 1, 1~
## $ scuba_fraction <dbl> 0.4, 1.0, 1.0, 0.2, 1.0, 0.2, 1.0, 0.4, 0.4, 0.2, 0.2, ~
## $ dist_type      <int> 0, 0, 0, 0, 0, 1, 0, 0, 0, 0, 0, 0, 0, 1, 0, 0, 0, 1, 0~
## $ dist_severity  <dbl> 0.00, 0.00, 0.50, 0.25, 0.00, 1.00, 0.25, 0.25, 0.00, 0~
## $ psgseats       <int> 1, 2, 1, 3, 1, 2, 2, 3, 8, 3, 5, 5, 2, 1, 1, 2, 5, 1, 2~
## $ divbl          <int> 0, 0, 0, 0, 0, 1, 0, 0, 0, 0, 0, 0, 0, 1, 0, 0, 0, 0, 0~
## $ chgsites       <int> 0, 0, 1, 1, 0, 0, 0, 0, 0, 1, 1, 1, 0, 1, 1, 0, 0, 1, 1~
## $ chgact         <int> 0, 0, 1, 0, 0, 1, 0, 0, 0, 0, 0, 0, 0, 1, 1, 0, 0, 0, 1~
## $ chgopmode      <int> 0, 0, 0, 0, 0, 1, 0, 0, 0, 0, 0, 1, 0, 1, 1, 0, 0, 0, 1~
## $ nrm            <int> 0, 0, 1, 1, 0, 0, 0, 1, 1, 1, 1, 0, 1, 1, 1, 0, 0, 0, 0~
## $ insurance      <int> 0, 0, 0, 0, 0, 0, 0, 0, 0, 0, 0, 0, 0, 0, 0, 0, 0, 0, 0~
## $ monitor        <int> 0, 0, 1, 1, 0, 0, 0, 0, 1, 1, 1, 1, 1, 0, 1, 0, 0, 1, 0~
## $ relief         <int> 0, 0, 0, 0, 0, 1, 0, 0, 0, 0, 0, 0, 0, 0, 0, 0, 0, 0, 0~
## $ support        <int> 0, 0, 0, 0, 0, 1, 0, 0, 0, 0, 0, 0, 0, 0, 0, 0, 0, 0, 0~
## $ co2            <int> 0, 0, 1, 1, 0, 0, 0, 0, 0, 1, 1, 1, 0, 0, 1, 0, 1, 0, 1~
## $ educate        <int> 0, 0, 0, 0, 0, 0, 0, 0, 0, 0, 1, 0, 0, 0, 0, 0, 0, 0, 1~
## $ none           <int> 1, 1, 0, 0, 1, 0, 1, 0, 0, 0, 0, 0, 0, 0, 0, 1, 0, 0, 0~
## $ topresponse    <chr> "none", "none", "chgsites", "nrm", "none", "relief", "n~
```

6 Response clusters

We now calculate the partial correlations between the adaptive responses that operators adopted in response to climate disturbances. These partial correlations reflect whether particular responses were more frequently implemented together than others. We used Spearman's Rank correlation because our responses are measures on a binary scale.

We found eight positive partial correlations between our individual adaptive responses that were significant at a p-level of 5% (Figure 1). Based on these significant associations, we decided to make some changes to

the a priori classification of adaptive response as proposed in Table 3. Most notably we decided to merge the adaptive responses of operational change, product diversification, and livelihood diversification into a combined adaptive response cluster linked to changes in 'operating model' because they were all linked to responses on the business and operational side. Compared to our a priori categorization, we classified 'spatial diversification' as a separate adaptation cluster because it was frequently implemented and not significantly associated with any of the other adaptive responses.

We found that the adaptive responses of 'monitoring (reefs and/or climate)' and 'restoration' were significantly correlated, although our a priori classification had defined monitoring as a protective measure. We used the monitoring and restoration responses as separate responses in our consequent analysis because these were each implemented by a relatively large fraction of operators. In accordance with our a priori classification, the adaptive responses of 'relief measures' and 'support-seeking' were significantly correlated.

Finally, one of the adaptive responses that was mentioned as other response by 16% of the participants was 'visitor education', i.e. informing and educating visitors about the causes and consequences of the climate disturbances. We merged the visitor education response with 'climate action' because they were significantly associated and because visitor education could potentially have an effect on future carbon emissions similar to a company taking climate action itself.

```
response_cor <- data[, c("divbl","relief", "support", "chgopmode", "chgact", "chgsites",
                          "educate", "monitor", "nrm", "co2")]

pcor(response_cor,method="spearman")
```

```
## $estimate
##                 divbl      relief     support   chgopmode       chgact
## divbl      1.000000000  0.24839999 -0.12827148  0.42408823  0.001626400
## relief     0.248399993  1.00000000  0.63844626  0.12717991 -0.291822740
## support   -0.128271480  0.63844626  1.00000000 -0.21227795  0.390251492
## chgopmode  0.424088233  0.12717991 -0.21227795  1.00000000  0.549039860
## chgact     0.001626400 -0.29182274  0.39025149  0.54903986  1.000000000
## chgsites   0.005038315  0.14303538  0.00696928  0.18759442  0.151111197
## educate    0.020557887 -0.31138278  0.21496514  0.16221237 -0.103631115
## monitor   -0.148753251 -0.01614644  0.02203716  0.09439283 -0.080956614
## nrm        0.284949069 -0.15535622  0.18466615 -0.10499157  0.007919571
## co2       -0.188620511  0.31327254 -0.31743868 -0.02361897  0.207770008
##               chgsites     educate     monitor          nrm         co2
## divbl     0.005038315  0.02055789 -0.14875325  0.284949069 -0.18862051
## relief    0.143035380 -0.31138278 -0.01614644 -0.155356220  0.31327254
## support   0.006969280  0.21496514  0.02203716  0.184666148 -0.31743868
## chgopmode 0.187594425  0.16221237  0.09439283 -0.104991568 -0.02361897
## chgact    0.151111197 -0.10363111 -0.08095661  0.007919571  0.20777001
## chgsites  1.000000000  0.10220349  0.27363079  0.100891471  0.04510476
## educate   0.102203489  1.00000000 -0.12344256 -0.077435633  0.31130733
## monitor   0.273630790 -0.12344256  1.00000000  0.364170673  0.26086092
## nrm       0.100891471 -0.07743563  0.36417067  1.000000000  0.21902950
## co2       0.045104757  0.31130733  0.26086092  0.219029501  1.00000000
##
## $p.value
##                 divbl       relief      support    chgopmode       chgact
## divbl     0.000000000 8.196356e-02 3.746750e-01 2.146388e-03 9.910563e-01
## relief    0.081963556 0.000000e+00 6.089877e-07 3.787870e-01 3.975336e-02
## support   0.374675014 6.089877e-07 0.000000e+00 1.388753e-01 5.082878e-03
## chgopmode 0.002146388 3.787870e-01 1.388753e-01 0.000000e+00 3.647562e-05
## chgact    0.991056295 3.975336e-02 5.082878e-03 3.647562e-05 0.000000e+00
```

```
## chgsites  0.972298730 3.217156e-01 9.616889e-01 1.920431e-01 2.948590e-01
## educate   0.887313512 2.772459e-02 1.338281e-01 2.603870e-01 4.738801e-01
## monitor   0.302545487 9.113848e-01 8.792632e-01 5.143789e-01 5.762371e-01
## nrm       0.044885494 2.813433e-01 1.991999e-01 4.680631e-01 9.564697e-01
## co2       0.189579128 2.674405e-02 2.468445e-02 8.706682e-01 1.476590e-01
##             chgsites    educate     monitor         nrm        co2
## divbl     0.97229873 0.88731351 0.302545487 0.044885494 0.18957913
## relief    0.32171558 0.02772459 0.911384812 0.281343276 0.02674405
## support   0.96168886 0.13382814 0.879263185 0.199199941 0.02468445
## chgopmode 0.19204308 0.26038701 0.514378923 0.468063063 0.87066824
## chgact    0.29485903 0.47388007 0.576237126 0.956469689 0.14765898
## chgsites  0.00000000 0.48002543 0.054505473 0.485710031 0.75577762
## educate   0.48002543 0.00000000 0.393068113 0.592990718 0.02776436
## monitor   0.05450547 0.39306811 0.000000000 0.009325288 0.06728969
## nrm       0.48571003 0.59299072 0.009325288 0.000000000 0.12645718
## co2       0.75577762 0.02776436 0.067289695 0.126457184 0.00000000
##
## $statistic
##                 divbl     relief     support  chgopmode      chgact   chgsites
## divbl      0.00000000  1.7766502 -0.89609343  3.2443703  0.01126805 0.03490691
## relief     1.77665021  0.0000000  5.74701646  0.8883419 -2.11381657 1.00127370
## support   -0.89609343  5.7470165  0.00000000 -1.5050049  2.93658877 0.04828576
## chgopmode  3.24437029  0.8883419 -1.50500487  0.0000000  4.55118111 1.32318334
## chgact     0.01126805 -2.1138166  2.93658877  4.5511811  0.00000000 1.05909087
## chgsites   0.03490691  1.0012737  0.04828576  1.3231833  1.05909087 0.00000000
## educate    0.14245933 -2.2701865  1.52497345  1.1389243 -0.72186408 0.71181395
## monitor   -1.04218777 -0.1118804  0.15271501  0.6569058 -0.56273097 1.97099306
## nrm        2.05956933 -1.0895684  1.30179375 -0.7314455  0.05487012 0.70258158
## co2       -1.33068699  2.2854583 -2.31923319 -0.1636827  1.47158623 0.31281329
##             educate    monitor         nrm        co2
## divbl      0.1424593 -1.0421878  2.05956933 -1.3306870
## relief    -2.2701865 -0.1118804 -1.08956844  2.2854583
## support    1.5249734  0.1527150  1.30179375 -2.3192332
## chgopmode  1.1389243  0.6569058 -0.73144554 -0.1636827
## chgact    -0.7218641 -0.5627310  0.05487012  1.4715862
## chgsites   0.7118139  1.9709931  0.70258158  0.3128133
## educate    0.0000000 -0.8618266 -0.53810555  2.2695774
## monitor   -0.8618266  0.0000000  2.70907454  1.8721169
## nrm       -0.5381055  2.7090745  0.00000000  1.5552450
## co2        2.2695774  1.8721169  1.55524499  0.0000000
##
## $n
## [1] 58
##
## $gp
## [1] 8
##
## $method
## [1] "spearman"
```

We then apply these clusters to our dataset before we start our analysis.

```
data_modified <- data %>%
  mutate(operational = ifelse(divbl + chgact + chgopmode > 0, 1, 0),
         coping = ifelse(relief + support + insurance > 0, 1, 0),
         climate = ifelse(co2 + educate > 0, 1, 0)
         )
```

7 Predictor data preparation

For the predictors, we transformed the age of the company representative into a binary predictor (older versus younger), the business type (snorkel vs. scuba), and the company size (# passenger seats on boats) into a categorical predictor. We measured the number of passenger seats using nine multiple-choice options that ranged from '0-10 seats' to '>500 seats'. Through visual inspection of the data, we identified three clusters that we consequently categorized as small (<20 seats), medium (20-200 seats), and large (>200 seats). We included company size as a categorical rather than an ordinal predictor because the effects were not ordered linearly for all response models. We used small-sized companies as the reference group.

Business type (scuba vs. snorkeling)

```
ggplot(data_modified , aes(x=scuba_fraction)) + ylab("Frequency") +
  geom_bar() + geom_vline(xintercept = 0.5) +
  annotate("text", x=0.4, y=30, label= "Snorkel") +
  annotate("text", x=0.6, y=30, label= "Scuba")
```

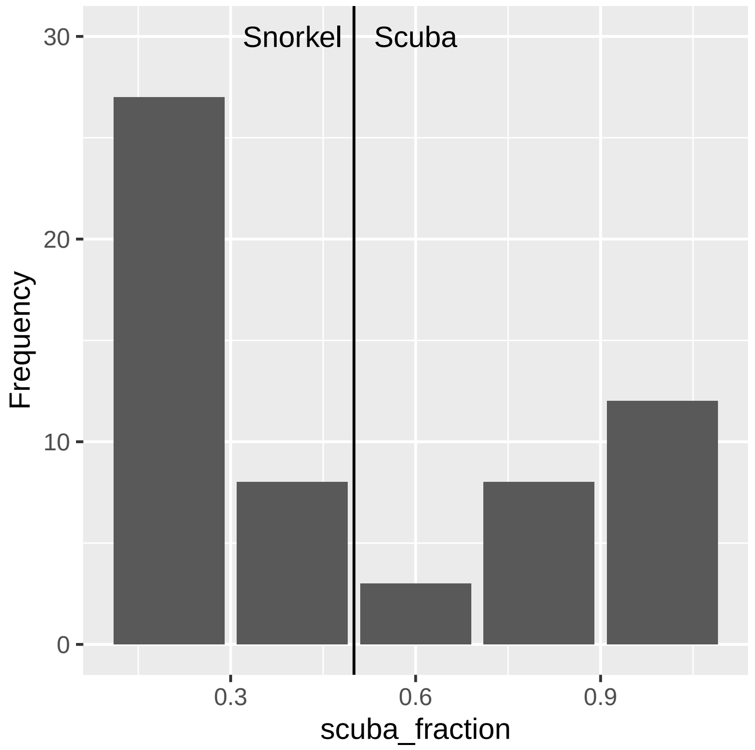

Business size (# passenger seats)

For boats ('psgseats'): 1 for 0 – 10 seats, 2 for 10 – 20 seats, 3 for 20 – 50 seats, 4 for 50 – 100 seats, 5 for 100 – 200 seats, 6 for 200 – 300 seats, 7 for 300 – 400 seats, 8 for 400 – 500 seats, and 9 for >500 seats.

```
ggplot(data_modified , aes(x=psgseats)) + ylab("Frequency") +
  geom_bar() + geom_vline(xintercept = 2.5) +
  geom_bar() + geom_vline(xintercept = 5.5) +
  annotate("text", x=1.5, y=20, label= "Small") +
  annotate("text", x=4, y=20, label= "Medium")+
  annotate("text", x=7, y=20, label= "Large")
```

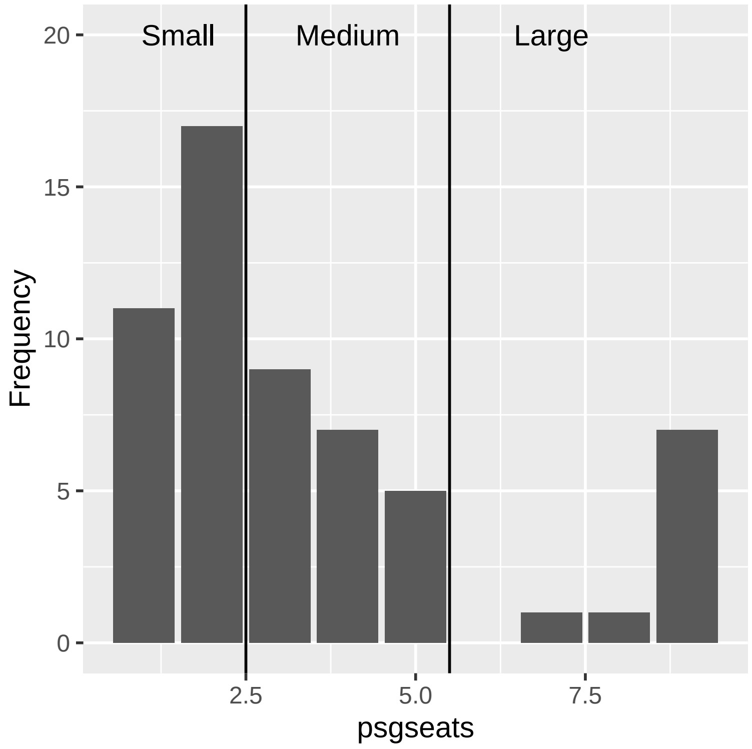

Representative gender

```
ggplot(data_modified , aes(x=resp_gender)) + ylab("Frequency") +
  geom_bar() + geom_vline(xintercept = 0.5) +
  annotate("text", x=0.2, y=35, label= "Male") +
  annotate("text", x=0.75, y=35, label= "Female")
```

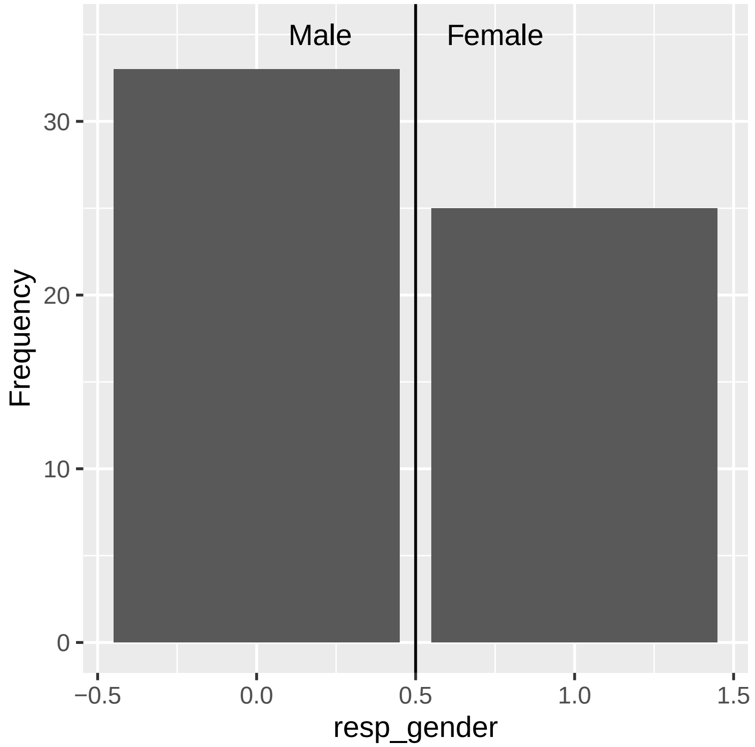

Representative age

```
ggplot(data_modified , aes(x=resp_age)) + ylab("Frequency") +
  geom_bar() + geom_vline(xintercept = 3.5) +
  annotate("text", x=2.5, y=22, label= "Younger") +
  annotate("text", x=4.5, y=22, label= "Older")
```

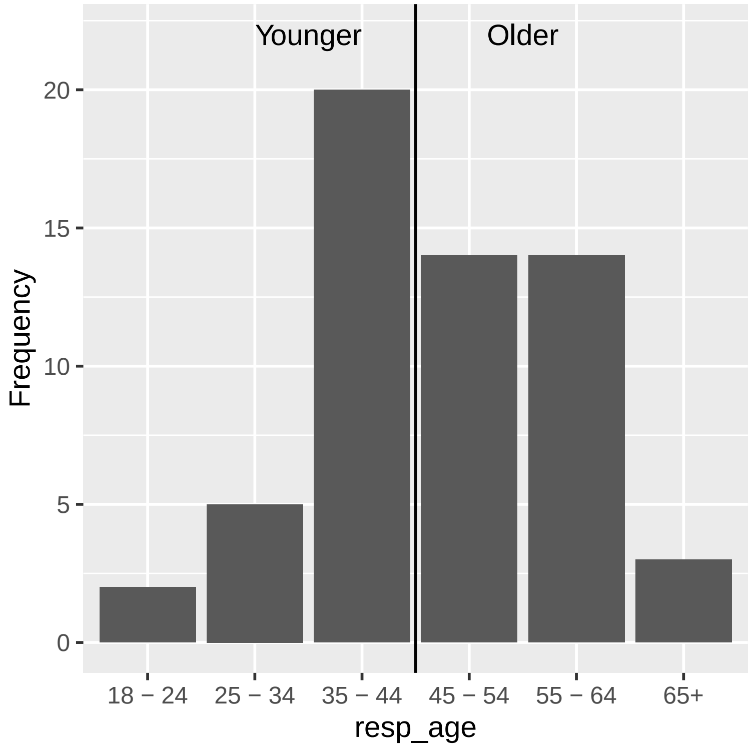

We now apply these modifications to our data using the code below.

Because all our predictors are on a binary scale, we standardized our only non-binary predictor (disturbance severity) using z-scores, by subtracting the mean and dividing by twice the standard deviation (Gelman, 2008). Dividing by twice the standard deviation standardizes each variable to have a mean of '0' and a standard deviation of '0.5'; this technically standardizes all predictors on a binary scale. Coefficients for continuous predictors from the Bayesian models are now directly comparable and should be interpreted as the effect of a one-standard deviation change in the predictor variable on the response variable.

```
data_modified <- data_modified %>%
  mutate(scuba_binary = ifelse(scuba_fraction > 0.4, 1, 0),
         resp_age_binary = case_when(
         resp_age == '18 - 24' | resp_age == '25 - 34'| resp_age == '35 - 44' ~ 1,
         resp_age == '45 - 54' | resp_age == '55 - 64'| resp_age == '65+' ~ 0),
         psgseats_cat = case_when(
         psgseats == 1 | psgseats == 2 ~ 'small',
         psgseats == 3 | psgseats == 4| psgseats == 5 ~ 'medium',
         psgseats == 6 | psgseats == 7| psgseats == 8| psgseats == 9 ~ 'large'),
         psgseats_cat = factor(psgseats_cat, levels = c('small', 'medium', 'large')),
         z.dist_severity =   (dist_severity-mean(dist_severity))/(2*sd(dist_severity))
         )

ggplot(data_modified, aes(x=scuba_binary)) +
  geom_bar()
```

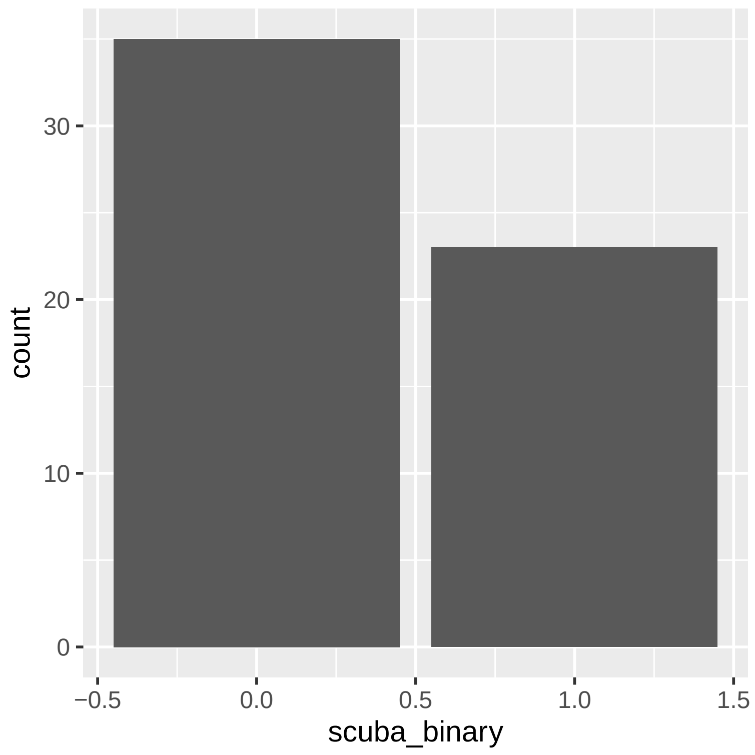


```
ggplot(data_modified, aes(x=resp_age_binary)) +
  geom_bar()
```

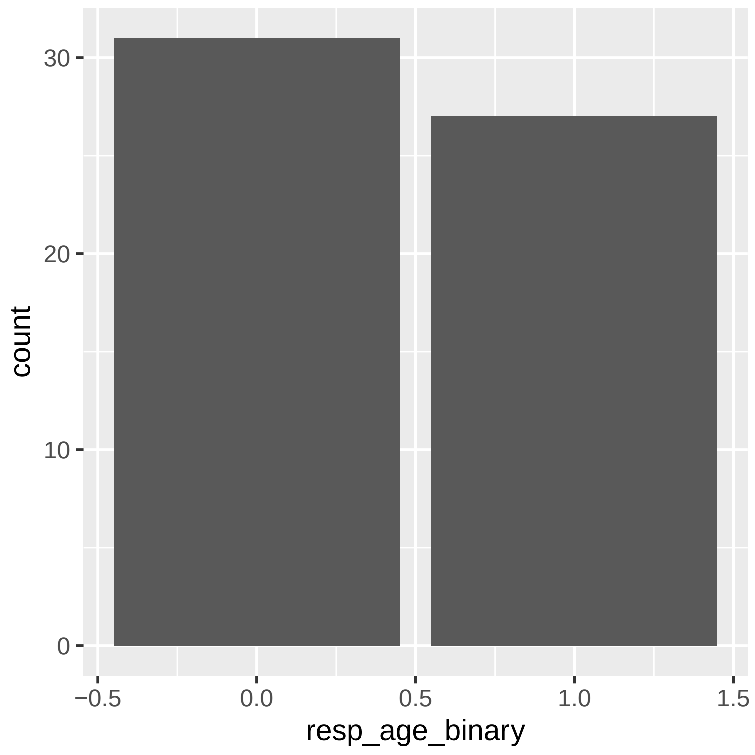

```
ggplot(data_modified, aes(x=psgseats_cat)) +
  geom_bar()
```

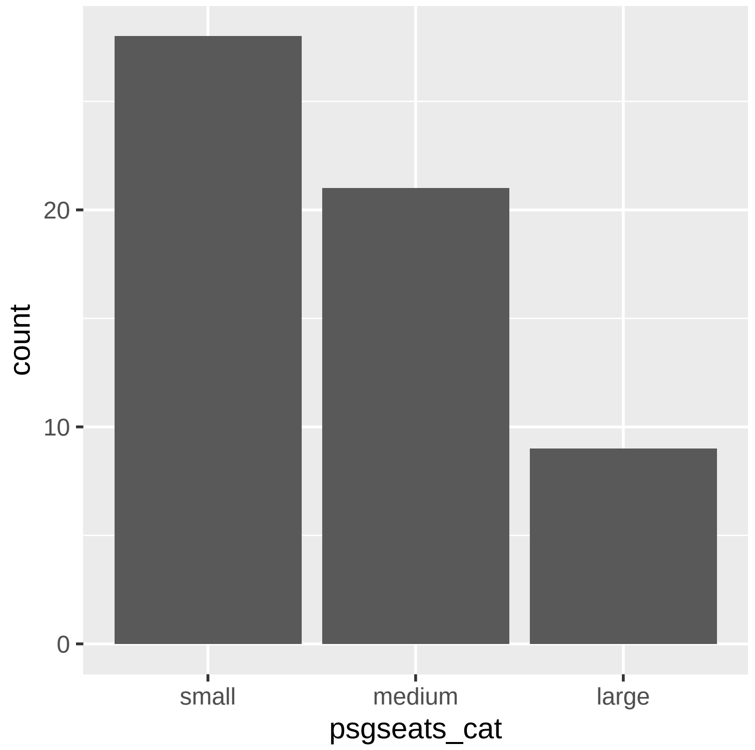


```
ggplot(data_modified, aes(x=z.dist_severity)) +
  geom_bar()
```

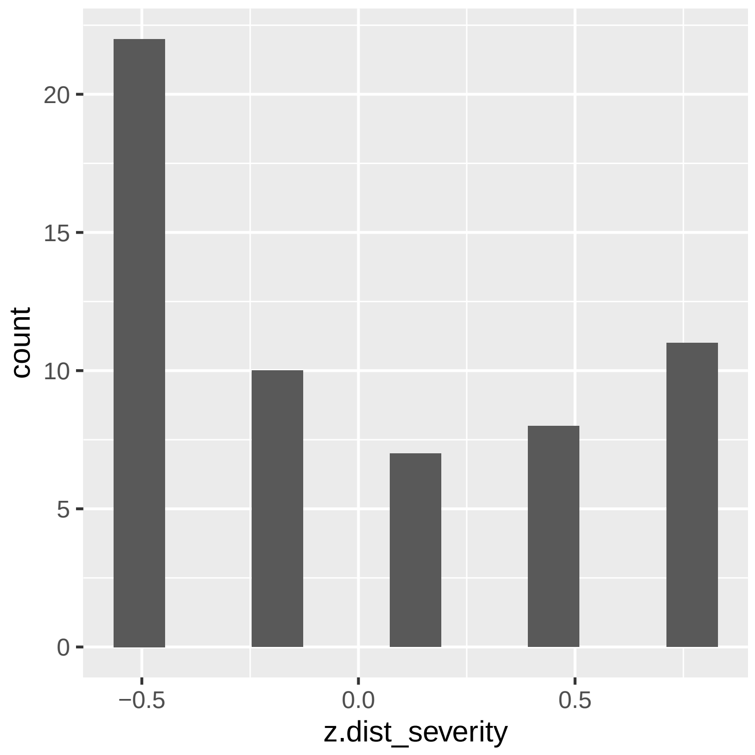

8 Analysis and model validation

Logistic regression model for changes in operating model response

Model definition and VIF test

```
operational.glm <- glm(operational ~ z.dist_severity + dist_type +
                   scuba_binary + psgseats_cat +
                   resp_age_binary + resp_gender,
                 data=data_modified, family=binomial(link='logit'))

check_collinearity(operational.glm)
```

```
## # Check for Multicollinearity
##
## Low Correlation
##
##             Term  VIF   VIF 95% CI Increased SE Tolerance Tolerance 95% CI
##  z.dist_severity 2.34 [1.75, 3.41]         1.53      0.43     [0.29, 0.57]
##        dist_type 2.02 [1.54, 2.92]         1.42      0.49     [0.34, 0.65]
##     scuba_binary 1.21 [1.05, 1.88]         1.10      0.82     [0.53, 0.95]
##     psgseats_cat 1.48 [1.20, 2.14]         1.22      0.68     [0.47, 0.83]
##  resp_age_binary 1.31 [1.10, 1.94]         1.15      0.76     [0.52, 0.91]
##      resp_gender 1.16 [1.03, 1.92]         1.08      0.86     [0.52, 0.97]
```

Model validation

```r
model.resid <- simulateResiduals(operational.glm)

plot(model.resid)
```

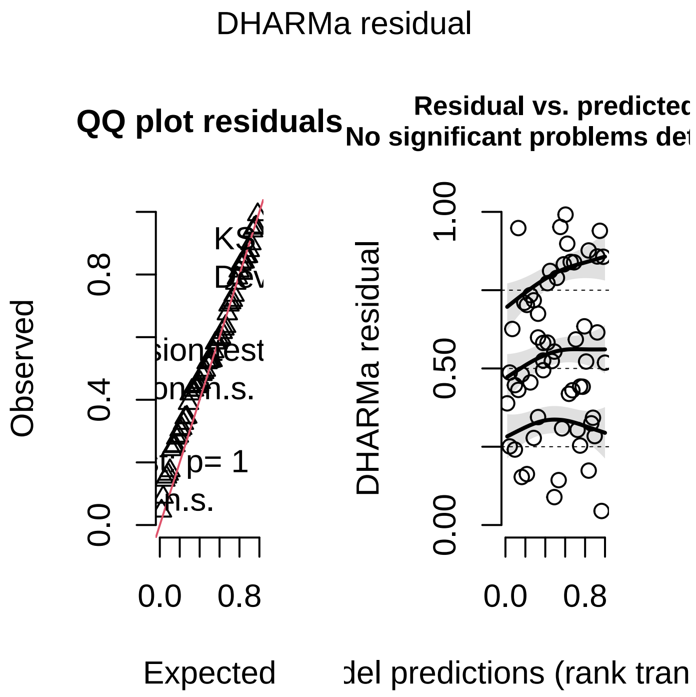

Model summary

```r
summary(operational.glm)
```

```
##
## Call:
## glm(formula = operational ~ z.dist_severity + dist_type + scuba_binary +
##     psgseats_cat + resp_age_binary + resp_gender, family = binomial(link = "logit"),
##     data = data_modified)
##
## Deviance Residuals:
##     Min       1Q   Median       3Q      Max
## -1.6688  -0.5682  -0.4287   0.6416   2.3081
##
## Coefficients:
##                     Estimate Std. Error z value Pr(>|z|)
## (Intercept)          -0.4891     0.8183  -0.598   0.5500
## z.dist_severity       2.8165     1.0967   2.568   0.0102 *
## dist_type            -0.2678     1.0174  -0.263   0.7924
## scuba_binary         -0.3218     0.7801  -0.412   0.6800
## psgseats_catmedium  -1.2541     0.8730  -1.437   0.1508
## psgseats_catlarge   -0.7447     1.0627  -0.701   0.4835
## resp_age_binary       0.1721     0.8022   0.215   0.8301
## resp_gender          -0.2770     0.7556  -0.367   0.7139
```

```
## ---
## Signif. codes:  0 '***' 0.001 '**' 0.01 '*' 0.05 '.' 0.1 ' ' 1
##
## (Dispersion parameter for binomial family taken to be 1)
##
##     Null deviance: 68.324  on 57  degrees of freedom
## Residual deviance: 52.911  on 50  degrees of freedom
## AIC: 68.911
##
## Number of Fisher Scoring iterations: 5
```

```
r2_operational <- 1 - (52.911 / 68.324)   #residual deviance / null deviance
r2_operational
```

```
## [1] 0.2255869
```

Logistic regression model for spatial diversification response

```
spatial.glm <- glm(chgsites ~ z.dist_severity + dist_type +
                    scuba_binary + psgseats_cat +
                    resp_age_binary + resp_gender,
                  data=data_modified, family=binomial(link='logit'))

check_collinearity(spatial.glm)
```

```
## # Check for Multicollinearity
##
## Low Correlation
##
##             Term  VIF   VIF 95% CI Increased SE Tolerance Tolerance 95% CI
##  z.dist_severity 1.88 [1.45, 2.71]         1.37      0.53     [0.37, 0.69]
##        dist_type 1.69 [1.33, 2.44]         1.30      0.59     [0.41, 0.75]
##     scuba_binary 1.09 [1.01, 2.36]         1.05      0.91     [0.42, 0.99]
##     psgseats_cat 1.17 [1.03, 1.91]         1.08      0.86     [0.52, 0.97]
##  resp_age_binary 1.35 [1.12, 1.98]         1.16      0.74     [0.51, 0.89]
##      resp_gender 1.40 [1.15, 2.04]         1.18      0.71     [0.49, 0.87]
```

Model validation

```
model.resid <- simulateResiduals(spatial.glm)

plot(model.resid)
```

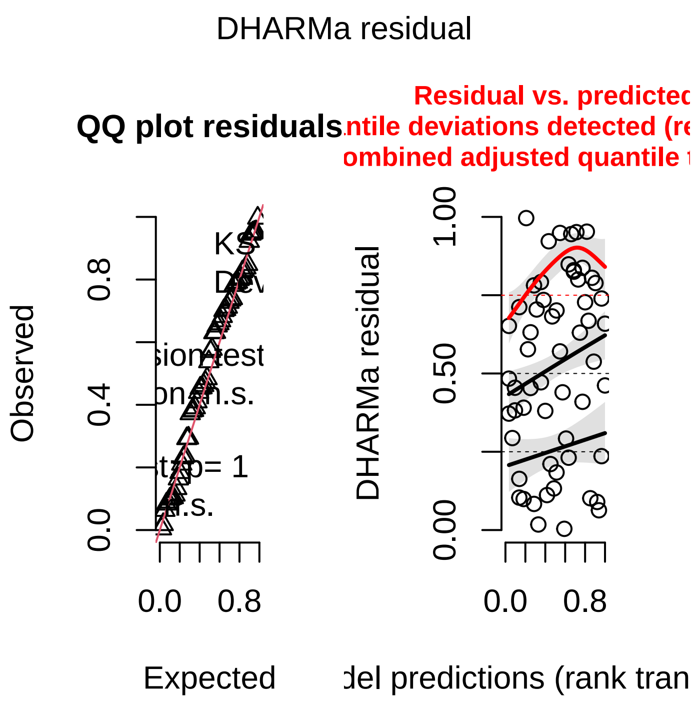

Model summary

```
summary(spatial.glm)
```

```
##
## Call:
## glm(formula = chgsites ~ z.dist_severity + dist_type + scuba_binary +
##     psgseats_cat + resp_age_binary + resp_gender, family = binomial(link = "logit"),
##     data = data_modified)
##
## Deviance Residuals:
##     Min       1Q   Median       3Q      Max
## -2.2507  -0.6511  -0.3915   0.6022   2.1689
##
## Coefficients:
##                      Estimate Std. Error z value Pr(>|z|)
## (Intercept)           -1.0299     0.8041  -1.281   0.2002
## z.dist_severity        1.9412     1.0604   1.831   0.0672 .
## dist_type              1.1131     1.1526   0.966   0.3342
## scuba_binary          -0.5180     0.7669  -0.675   0.4994
## psgseats_catmedium     0.9663     0.7981   1.211   0.2260
## psgseats_catlarge     -0.0305     1.0529  -0.029   0.9769
## resp_age_binary       -0.9604     0.8251  -1.164   0.2444
## resp_gender            1.0192     0.8382   1.216   0.2240
## ---
## Signif. codes:  0 '***' 0.001 '**' 0.01 '*' 0.05 '.' 0.1 ' ' 1
##
## (Dispersion parameter for binomial family taken to be 1)
##
```

```
##     Null deviance: 76.992  on 57  degrees of freedom
## Residual deviance: 50.789  on 50  degrees of freedom
## AIC: 66.789
##
## Number of Fisher Scoring iterations: 5
```

```r
r2_spatial <- 1 - (50.789 / 76.992)      #residual deviance / null deviance
r2_spatial
```

```
## [1] 0.3403341
```

Logistic regression model for monitoring response

```r
monitor.glm <- glm(monitor ~ z.dist_severity + dist_type +
                   scuba_binary + psgseats_cat +
                   resp_age_binary + resp_gender,
                 data=data_modified, family=binomial(link='logit'))

check_collinearity(monitor.glm)
```

```
## # Check for Multicollinearity
##
## Low Correlation
##
##             Term  VIF   VIF 95% CI Increased SE Tolerance Tolerance 95% CI
##  z.dist_severity 2.37 [1.77, 3.45]         1.54      0.42     [0.29, 0.57]
##        dist_type 2.28 [1.71, 3.30]         1.51      0.44     [0.30, 0.59]
##     scuba_binary 1.23 [1.06, 1.88]         1.11      0.81     [0.53, 0.94]
##     psgseats_cat 1.17 [1.03, 1.91]         1.08      0.86     [0.52, 0.97]
##  resp_age_binary 1.36 [1.13, 1.99]         1.17      0.73     [0.50, 0.88]
##      resp_gender 1.21 [1.05, 1.88]         1.10      0.82     [0.53, 0.95]
```

Model validation

```r
model.resid <- simulateResiduals(monitor.glm)

plot(model.resid)
```

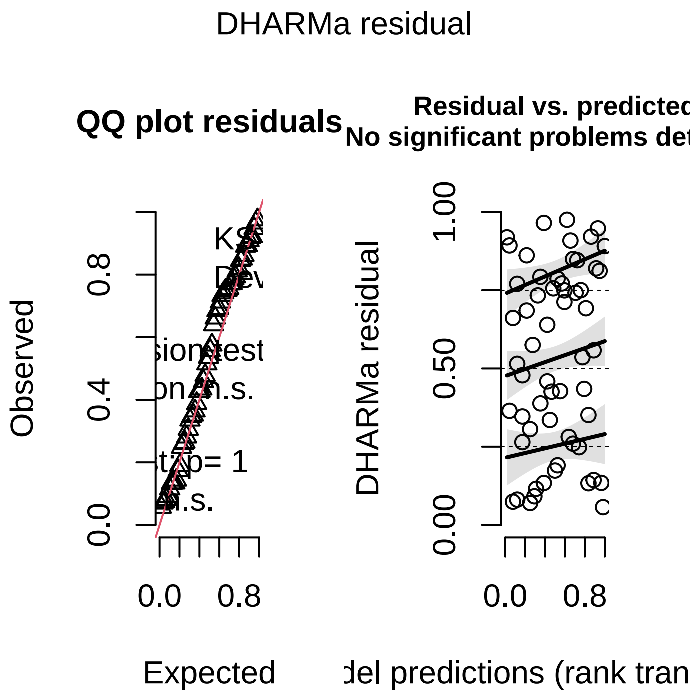

Model summary

```
summary(monitor.glm)
```

```
##
## Call:
## glm(formula = monitor ~ z.dist_severity + dist_type + scuba_binary +
##     psgseats_cat + resp_age_binary + resp_gender, family = binomial(link = "logit"),
##     data = data_modified)
##
## Deviance Residuals:
##     Min       1Q   Median       3Q      Max
## -2.0911  -0.9425   0.5485   0.9662   1.7773
##
## Coefficients:
##                     Estimate Std. Error z value Pr(>|z|)
## (Intercept)         -0.37854    0.71545  -0.529   0.5967
## z.dist_severity      1.91728    0.95157   2.015   0.0439 *
## dist_type           -0.71252    1.07601  -0.662   0.5079
## scuba_binary         0.44154    0.65833   0.671   0.5024
## psgseats_catmedium   0.91491    0.67280   1.360   0.1739
## psgseats_catlarge    0.90931    0.90901   1.000   0.3171
## resp_age_binary      0.06033    0.67702   0.089   0.9290
## resp_gender          0.11040    0.64284   0.172   0.8636
## ---
## Signif. codes:  0 '***' 0.001 '**' 0.01 '*' 0.05 '.' 0.1 ' ' 1
##
## (Dispersion parameter for binomial family taken to be 1)
##
```

```
##     Null deviance: 80.129  on 57  degrees of freedom
## Residual deviance: 69.348  on 50  degrees of freedom
## AIC: 85.348
##
## Number of Fisher Scoring iterations: 4
```

```r
r2_monitor <- 1 - (69.348 / 80.129)      #residual deviance / null deviance
r2_monitor
```

```
## [1] 0.1345455
```

Logistic regression model for restoration response

```r
nrm.glm <- glm(nrm ~ z.dist_severity + dist_type +
                   scuba_binary + psgseats_cat +
                   resp_age_binary + resp_gender,
                 data=data_modified, family=binomial(link='logit'))

check_collinearity(nrm.glm)
```

```
## # Check for Multicollinearity
##
## Low Correlation
##
##              Term  VIF   VIF 95% CI Increased SE Tolerance Tolerance 95% CI
##   z.dist_severity 3.79 [2.70, 5.61]         1.95      0.26     [0.18, 0.37]
##         dist_type 3.13 [2.26, 4.60]         1.77      0.32     [0.22, 0.44]
##      scuba_binary 1.21 [1.05, 1.88]         1.10      0.83     [0.53, 0.95]
##      psgseats_cat 1.23 [1.06, 1.88]         1.11      0.81     [0.53, 0.94]
##   resp_age_binary 1.76 [1.37, 2.53]         1.33      0.57     [0.40, 0.73]
##       resp_gender 1.50 [1.21, 2.17]         1.22      0.67     [0.46, 0.82]
```

Model validation

```r
model.resid <- simulateResiduals(nrm.glm)

plot(model.resid)
```

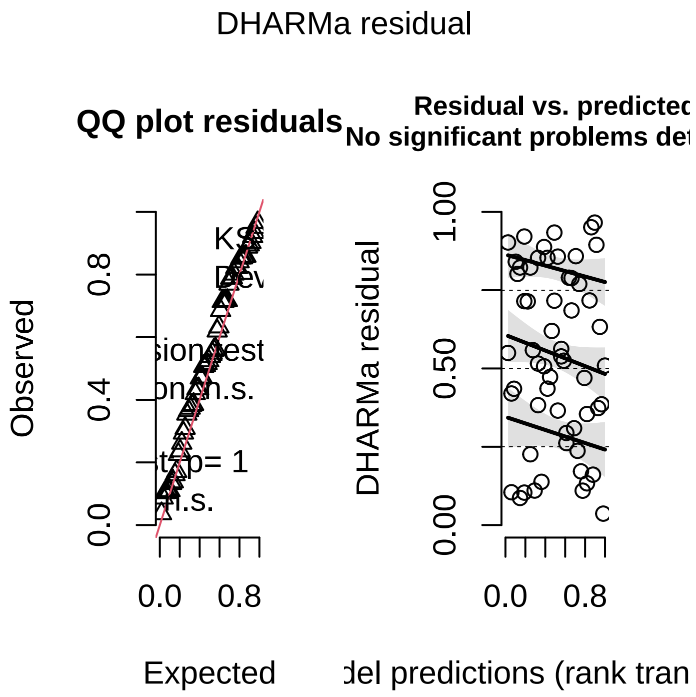

Model summary

```
summary(nrm.glm)
```

```
##
## Call:
## glm(formula = nrm ~ z.dist_severity + dist_type + scuba_binary +
##     psgseats_cat + resp_age_binary + resp_gender, family = binomial(link = "logit"),
##     data = data_modified)
##
## Deviance Residuals:
##     Min       1Q   Median       3Q      Max
## -1.7498  -0.7521  -0.3551   0.6881   2.2851
##
## Coefficients:
##                      Estimate Std. Error z value Pr(>|z|)
## (Intercept)            0.2975     0.8547   0.348  0.72779
## z.dist_severity        4.1055     1.3922   2.949  0.00319 **
## dist_type             -2.7922     1.3456  -2.075  0.03798 *
## scuba_binary          -0.7549     0.7841  -0.963  0.33569
## psgseats_catmedium     0.6210     0.7973   0.779  0.43605
## psgseats_catlarge      1.4673     1.0578   1.387  0.16540
## resp_age_binary        1.3234     0.8990   1.472  0.14097
## resp_gender           -2.1158     0.8485  -2.493  0.01265 *
## ---
## Signif. codes:  0 '***' 0.001 '**' 0.01 '*' 0.05 '.' 0.1 ' ' 1
##
## (Dispersion parameter for binomial family taken to be 1)
##
```

```
##     Null deviance: 78.672  on 57  degrees of freedom
## Residual deviance: 53.795  on 50  degrees of freedom
## AIC: 69.795
##
## Number of Fisher Scoring iterations: 5
```

```r
r2_nrm <- 1 - (53.795 / 78.672)      #residual deviance / null deviance

r2_nrm
```

```
## [1] 0.3162116
```

Logistic regression model for climate action

```r
climate.glm <- glm(climate ~ z.dist_severity + dist_type +
                   scuba_binary + psgseats_cat +
                   resp_age_binary + resp_gender,
                 data=data_modified, family=binomial(link='logit'))

check_collinearity(climate.glm)
```

```
## # Check for Multicollinearity
##
## Low Correlation
##
##             Term  VIF   VIF 95% CI Increased SE Tolerance Tolerance 95% CI
##  z.dist_severity 2.21 [1.67, 3.21]         1.49      0.45     [0.31, 0.60]
##        dist_type 2.17 [1.64, 3.14]         1.47      0.46     [0.32, 0.61]
##     scuba_binary 1.27 [1.08, 1.91]         1.13      0.79     [0.52, 0.93]
##     psgseats_cat 1.31 [1.10, 1.93]         1.14      0.77     [0.52, 0.91]
##  resp_age_binary 1.33 [1.11, 1.95]         1.15      0.75     [0.51, 0.90]
##      resp_gender 1.19 [1.04, 1.89]         1.09      0.84     [0.53, 0.96]
```

Model validation

```r
model.resid <- simulateResiduals(climate.glm)

plot(model.resid)
```

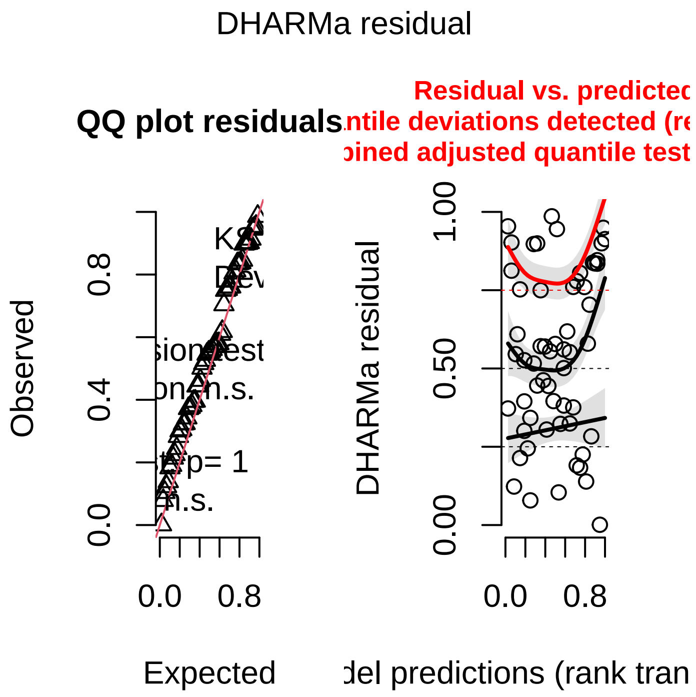

Model summary

```
summary(climate.glm)
```

```
##
## Call:
## glm(formula = climate ~ z.dist_severity + dist_type + scuba_binary +
##     psgseats_cat + resp_age_binary + resp_gender, family = binomial(link = "logit"),
##     data = data_modified)
##
## Deviance Residuals:
##     Min       1Q   Median       3Q      Max
## -1.7764  -0.8489  -0.5892   0.9329   2.1973
##
## Coefficients:
##                      Estimate Std. Error z value Pr(>|z|)
## (Intercept)          -0.21515    0.72096  -0.298   0.7654
## z.dist_severity       1.26144    0.88851   1.420   0.1557
## dist_type            -1.26296    1.03177  -1.224   0.2209
## scuba_binary          0.99290    0.68373   1.452   0.1465
## psgseats_catmedium    0.67397    0.70596   0.955   0.3397
## psgseats_catlarge     1.06498    0.95577   1.114   0.2652
## resp_age_binary      -1.46698    0.69895  -2.099   0.0358 *
## resp_gender           0.07798    0.66156   0.118   0.9062
## ---
## Signif. codes:  0 '***' 0.001 '**' 0.01 '*' 0.05 '.' 0.1 ' ' 1
##
## (Dispersion parameter for binomial family taken to be 1)
##
```

```
##     Null deviance: 79.298  on 57  degrees of freedom
## Residual deviance: 66.292  on 50  degrees of freedom
## AIC: 82.292
##
## Number of Fisher Scoring iterations: 4
```

```r
r2_climate <- 1 - (66.292 / 79.298)      #residual deviance / null deviance

r2_climate
```

```
## [1] 0.1640142
```
